# Myocardial Ischemic Subject’s Thymus Fat: A Novel Source of Multipotent Stromal Cells

**DOI:** 10.1371/journal.pone.0144401

**Published:** 2015-12-10

**Authors:** Wilfredo Oliva-Olivera, Leticia Coín-Aragüez, Julián Salas, Said Lhamyani, Adriana-Mariel Gentile, Esteban Sarria García, Abdelkrim Hmadcha, Hatem Zayed, Antonio Vega-Rioja, Francisco J. Tinahones, Rajaa El Bekay

**Affiliations:** 1 CIBER Pathophysiology of obesity and nutrition CB06/03, Carlos III Health Institute, Malaga, 29010, Spain, Laboratory of Biomedical Research, Virgen de la Victoria Clinical University Hospital, Málaga, 29010, Spain; 2 Cardiovascular Surgery Department, University Hospital Carlos Haya, Málaga, Spain; 3 Research Laboratory, Science School, Málaga University, Campus Teatinos s/n– 29071, Málaga, Spain; 4 Andalusian Center for Molecular Biology and Regenerative Medicine (CABIMER), Department of Stem Cells, Sevilla, Spain, Centro de Investigación Biomédica en Red-Diabetes y Enfermedades Metabólicas (CIBERDEM), Instituto de Salud Carlos III, Madrid, Spain; 5 Biomedical Sciences Program, Health Sciences Department, College of Arts and Sciences, Qatar University, P.O. Box: 2713, Doha, Qatar; 6 Servicio Regional de Inmunología y Alergia, Hospital Universitario Virgen Macarena, Sevilla, Spain; French Blood Institute, FRANCE

## Abstract

**Objective:**

Adipose Tissue Stromal Cells (ASCs) have important clinical applications in the regenerative medicine, cell replacement and gene therapies. Subcutaneous Adipose Tissue (SAT) is the most common source of these cells. The adult human thymus degenerates into adipose tissue (TAT). However, it has never been studied before as a source of stem cells.

**Material and Methods:**

We performed a comparative characterization of TAT-ASCs and SAT-ASCs from myocardial ischemic subjects (n = 32) according to the age of the subjects.

**Results:**

TAT-ASCs and SAT-ASCs showed similar features regarding their adherence, morphology and in their capacity to form CFU-F. Moreover, they have the capacity to differentiate into osteocyte and adipocyte lineages; and they present a surface marker profile corresponding with stem cells derived from AT; CD73^+^CD90^+^CD105^+^CD14^-^CD19^-^CD45^-^HLA-DR. Interestingly, and in opposition to SAT-ASCs, TAT-ASCs have CD14^+^CD34^+^CD133^+^CD45^-^ cells. Moreover, TAT-ASCs from elderly subjects showed higher adipogenic and osteogenic capacities compared to middle aged subjects, indicating that, rather than impairing; aging seems to increase adipogenic and osteogenic capacities of TAT-ASCs.

**Conclusions:**

This study describes the human TAT as a source of mesenchymal stem cells, which may have an enormous potential for regenerative medicine.

## Introduction

Mesenchymal stem cells are a heterogeneous population of stem cells capable of self-renewing and differentiating into osteoblasts, chondrocytes, adipocytes, myocytes, cardiomyocytes, fibroblasts, myofibroblasts, epithelial and neural cells [1]. These unique properties make them of great interest for tissue engineering and regenerative medicine [2]. Although they are found mainly in the bone marrow, they can also be found in the Adipose Tissue (AT), peripheral blood, umbilical cord, liver, and foetal tissues, among others. Once isolated, they have been cultured which has allowed studying their phenotypic and functional features [3,4].

Several studies have found that AT is a feasible abundant source of mesenchymal stem cells for regenerative medicine [5] and that these cells can be isolated in a reliable and reproducible manner [6] in comparison to mesenchymal stem cells from bone marrow [7].

Given that mesenchymal stem cells have considerable therapeutic potential, and have generated markedly increasing interest in a wide variety of biomedical disciplines, The Mesenchymal and Tissue Stem Cell Committee of the International Society for Cellular Therapy proposes minimal criteria to define human mesenchymal stem cells [8]: 1) These cells must be plastic-adherent when maintained in standard culture conditions; 2) They must express CD105, CD73 and CD90, and lack expression of CD45, CD34, CD14 or CD11b, CD79a or CD19 and HLA-DR surface molecules; 3) They must differentiate to osteoblasts, adipocytes and chondroblasts *in vitro*.

The thymus is largest and most active during the neonatal and pre-adolescent periods. By the early teens, the thymus begins to atrophy and thymic stroma is mostly replaced by mature AT [9]. Previous studies have highlighted the adult thymus as a potential source of ASCs [10–12]. Our group has been investigating the potential use of thymus fat as a source for cellular therapy for heart neovascularization after cardiopulmonary bypass interventions (CPB) on myocardial ischemic elderly subjects. These subjects cannot benefit from coronary revascularization treatment after surgery or angioplasty. Part of the thymus fat is discarded in aortic cannulation procedures of the ascending aorta in cardiovascular surgery in patients with a need of a CPB. Therefore, it is an available source of TAT. We have previously described that adult thymus fat from myocardial ischemic subjects presents a similar cellular profile as that observed in other white adipose tissues such as SAT. This cellular variety seems to be responsible for the observed differences in the expression of a variety of genes that are known to be relevant in the regulation of the neo genesis of adipocytes and of other cell types such as endothelial [13]. These data suggested that it would be relevant to analyze whether TAT is a source of mesenchymal stem cells and to compare the characteristics of these cells with those from SAT.

## Materials and Methods

### Patients and adipose tissue collection

The study was performed conform the declaration of Helsinki and recruited myocardial ischemic patients (n = 32) who received a coronary-artery bypass graft with a CPB in the Carlos Haya Hospital. The mean number of grafts used was 3.1 per patient. The subjects operated on and recruited in this study were individuals with stable situations and without severe ischemic injury. Therefore, these subjects were without infarct or with a previous infarct at least 6 months before surgery. For the stromal vascular fraction (SVF) cell proliferation assay, Fibroblast Colony Forming Units assay (CFU-F assay) and flow cytometry study, the number of patients with similar clinical characteristics was 6 (all of them with an age ≥ 70 years). The differentiation assays and mRNA expression analysis were performed on two groups of patients: the middle-aged group, aged from 45, and the elderly group of patients with myocardial ischemia. The number of patients recruited for the study of adipogenic and osteogenic differentiation was 26 (n = 8 middle aged, and n = 18 elderly). The local ethical committee of the Carlos Haya Hospital approved this study, and the Spanish Society of Thoracic and Cardiovascular Surgery (SECTCV) obtained signed informed consent from all participating patients. The relevant clinical and metabolic characteristics of these subjects are shown in Table 1. Both SAT and TAT were obtained at the beginning of the procedure and before heart arrest. The site of SAT harvest was from the chest incision. Fresh tissues were immediately processed for mesenchymal cell extraction and flow cytometry or characterization.

**Table 1:** Clinical variables of Myocardial Ischemic Subjects (n = 32).

Parameters	Middle- aged (n = 18)	Elderly (n = 18)	P
**Age (years)**	45–65	≥ 70	
**Triglycerides (mg/dL)**	149.00 ± 20.67	129.0 ±20.72	NS
**Cholesterol (mg/dL)**	180.50 ±12.92	183.92±8.48	NS
**HDL-c (mg/dL)**	50.63 ± 8.86	49.08±3.34	NS
**LDL-c (mg/dL9**	101.58 ± 10.86	109.03±6.28	NS
**Creatinine (mg/dL)**	1.085 ± 0.14	1.36±0.36	NS
**Hb1Ac(%)**	13.33 ±0.67	12.31±0.42	NS
**Hematocrit (%)**	40.53 ±1.87	37.97±1.35	NS
**BMI (kg/m** ^**2**^ **)**	30.59 ±1.19	28.08±0.88	NS

Values are means ± SEM. Comparisons between both groups were carried out using the Mann-Whitney U Test. HDL-C: Cholesterol HDL, LDL-c: Cholesterol LDL, BMI: Body Mass Index, NS: No significant.

### Isolation and expansion of cells from the SVF of TAT and SAT

Isolation and expansion of cells from SVF was performed as previously described [14] with modifications. Briefly, both SAT and TAT were obtained and transported in Hank's balanced salt solution supplemented with penicillin and streptomycin. AT biopsies were washed with PBS, dissected into smaller pieces under sterile conditions, and subjected to enzymatic digestion with a solution containing 0.150% collagenase type I and 1.0% bovine serum albumin (BSA) for 70 min at 37°C. The cell suspension was centrifuged at 500 x *g* for 10 min. Floating adipocytes were discarded and the pellet containing the SVF was filtered through a 100-μm mesh, and centrifuged at 400×*g* for 5 min. The cell pellets were re-suspended in erythrocyte lysis buffer for 10 min at room temperature and centrifuged at 400 x *g* for 5 min. Cell pellets were then suspended in expansion medium DMEM/F12 supplemented with 10% fetal bovine serum, 100 μg/ml streptomycin, 100 U/ml penicillin, 1 μg/ml amphotericin B and 2 mM L-glutamine. Cells were them plated in tissue culture flasks and incubated at 37°C in a humid atmosphere with 5% of CO_2_ for approximately 8 days until 90% confluence was reached. The cells were always used between passages one/three.

### SVF Cell proliferation assay

Cells from the SVF from each donor (n = 6) were seeded in triplicate in 12 well plates at 5000 cells per cm^2^ in complete expansion medium. Cells were dissociated by trypsin and counted every 48 hours for 23 days using the trypan blue exclusion method.

### Population doubling assay

5000 ASCs from SAT and TAT of each donor (n = 6) were seeded in triplicate on 12 well plates. The cells were cultured until reaching confluence, dissociated by trypsin, and counted using the trypan blue exclusion method. The population doublings (PDs) were calculated using the following equation: PDs = 240/Log2 (N2/N1), where N1 and N2 represent the average cell number at 5^th^ and 15^th^ day, respectively.

### Colony Forming Unit-Fibroblastic (CFU-F) assay

Cells from the SVF of each donor (n = 6) were seeded in triplicate in 6 well plates at 50 cells per cm^2^. The cells were cultured for 14 days under standard conditions (37°C in a 5% CO_2_ moist atmosphere). At day 14, medium was removed and resultant colonies were washed twice with PBS, fixed with absolute methanol and stained with 0.5% crystal violet for 20 minutes at room temperature. The plates were washed with water, and colonies containing more than 50 cells were counted.

### Immunophenotypic characterization by flow cytometry

Cells from the SVF at passage 3 were immunophenotyped by flow cytometry using cell surface markers CD14, CD34, CD45, CD73, HLA-DR (BD Pharmigen, EEUU), CD29, CD31, CD44, CD49D, CD19, CD90, CD105, CD106, CD133, CD144, CD146, (eBioscience), CD140A, CD140B, CD166 (RD Systems, EEUU). The clone, fluorochrome and amount of each antibody are provided in S1 Table. Briefly, 10^6^cells/ml were resuspended in blocking buffer solution containing PBS supplemented with 3.0% BSA, and incubated on ice for 10 minutes. Then, they were gated according to their granularity (SS) and size (FS). 1x10^5^ cells were stained using the antibodies (0.25 μg x 10^6^ cells in 100 μl PBS) against the above surface markers or the isotype-matched control antibody. Samples were analyzed on a FACScan CyAnTM High-speed ADP Analyzer (Beckman Coulter, CA. EEUU). Data acquisition and analysis were performed using SUMMIT 3 software (Beckman Coulter). Unstained cells were used to establish flow cytometer settings. Debris and cells/particles with auto-fluorescence were removed by using a threshold on the forward scatter. The original (RAW) set of data is shown in S1 Fig.

### In vitro adipogenic and osteogenic differentiation assay

Cells from SVF of TAT and SAT were harvested at passage 3 and seeded in 6 well plates at 10,000 cells per cm^2^ in expansion medium until reaching 90% confluence. The expansion medium was changed every 2–3 days, and replaced with adipogenic or osteogenic medium at day 14.

The adipogenic induction medium was DMEM/F12 supplemented with 10% FBS, streptomycin 100 μg/ml, penicillin 100 U/ml, L-glutamine 2 mM, insulin 10 μM, isobutilmetilxantine 0.5 mM, dexamethasone 1.0 μM, pioglitazone 10 μM, rosiglitazone 0.5 μM, biotin 33 μM, and pathenonate 17 μM. After 72h of adipogenic induction, the medium was replaced by the above medium without Biotin and Pathenonate. Adipogenesis was confirmed after 14 days by oil Red O staining to visualize the characteristic cytoplasmic lipid droplets.

The osteogenic differentiation was performed with DMEM/F12 containing FBS 10%, streptomycin 100 μg/mL, penicillin 100 U/mL, L-glutamine 2 mM, dexamethasone 1.0 μM, ascorbic acid 200 μM, and β-glycerolphosphate 20 mM. The osteogenic potential was evaluated by assessment of calcium deposition by Alizarin Red S staining.

### Quantitative reverse transcriptase polymerase chain reaction (RT-PCR) analysis

Total RNA was isolated using the RNA-Stat 60 Reagent (Ams Biotechnology, UK). The amplifications were performed using a MicroAmp optical 96-well reaction plate (PE Applied Biosystems) on an ABI 7500 real-time PCR system (Applied Biosystems). RT qPCR reactions were carried out for all genes using specific TaqMan gene expression assays. During PCR, the Ct values for each amplified product were determined using a threshold value of 0.1. The specific signals were normalized by constitutively expressed cyclophilin (4326316E) signals according to the formula 2^−ΔCt^.

TaqMan® gene expression assay probes:

*SREBP1*          Hs00967385_g1

*FABP-4*            Hs01086177_m1

*LPL*                  Hs00173425_m1

*FASN*              Hs00188012_m1

*ADRP*              Hs00765634_m1

*CEBPα*            Hs00269972_s1

*ALPL*              Hs01029144_m1

### Statistical analysis

The results are given as mean and standard errors mean (±SEM). All clinical parameters are summarized by descriptive statistics (Table 1). Relationships between TAT and SAT, and between the elderly and middle-aged groups were performed using Mann-Whitney U test. Relationships between control wells and differentiation wells were performed using a signed rank test (Wilcoxon). Correlation analysis was performed using a Spearman’s correlation coefficient test (*r*). In all cases, the rejection level for a null hypothesis was α = 0.05 for two tails. The statistical analysis was carried out with the SPSS software program (Version 15.0 for Windows; SPSS, Chicago, IL).

## Results

### Clinical and biological variables of both patient groups

Table 1 shows that there were no significant differences in clinical and biological variables between the two groups.

### Comparative analysis of the ability to adhere to plastic and form CFU-F of TAT-ASCs and SAT-ASCs

**Fig 1:**
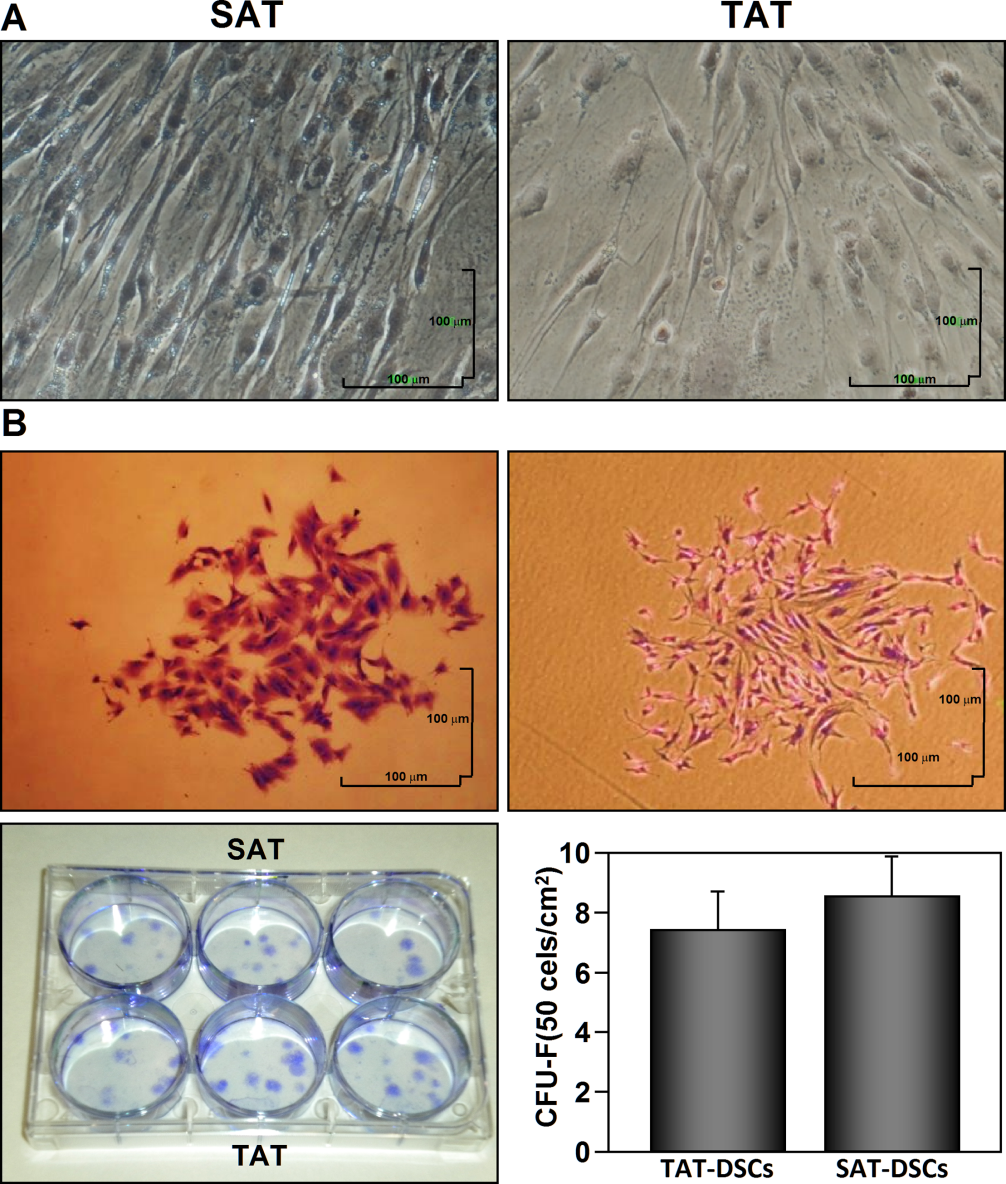
Comparison of colony forming units–fibroblast (CFU-Fs) between human thymus and subcutaneous adipose tissue-derived stromal cells (TAT-ASCs and SAT-ASCs). TAT and SAT derived- stromal cells (TAT-ASCs and SAT-ASCs) were extracted from the tissues of myocardial ischemic subjects (n = 6) and were seeded in triplicate in 6 well plates at 50 cells/cm^2^. The cells were cultured for 14 days under standard conditions (37°C in a 5% CO2 moist atmosphere). At day 14 cells were stained with crystal violet and colonies containing more than 50 cells were counted. (A) Microscopic image of adhered cells to plastic dishes. (B) Microscopic image of representative colony of each cell type (TAT-ASCs and SAT-ASCS). The results are representative of four independent experiments. Scale bars = 100 μm. *P <0.05. The graph bar represents mean ± SEM. The comparison between the two ASC types were carried out with Mann-Whitney U-Test. Image magnification is 4X.

SAT-ASCs and TAT-ASCs showed a similar ability to adhere to plastic culture plates, and displayed elongated and flattened fibroblastic morphology, a morphology compatible with clonogenic cell colonies, the characteristics of human Mesenchymal Stem Cells (Fig 1A). A CFU-F assay was performed in order to quantify the differences between TAT-ASCs and SAT-ASCs. As shown in Fig 1B, no significant differences were observed between TAT-ASCs (7.45±1.29) and SAT-SCs (8.55±1.42).

### Comparative analysis of the growth kinetics of TAT-ASCs and SAT-ASCs

**Fig 2:**
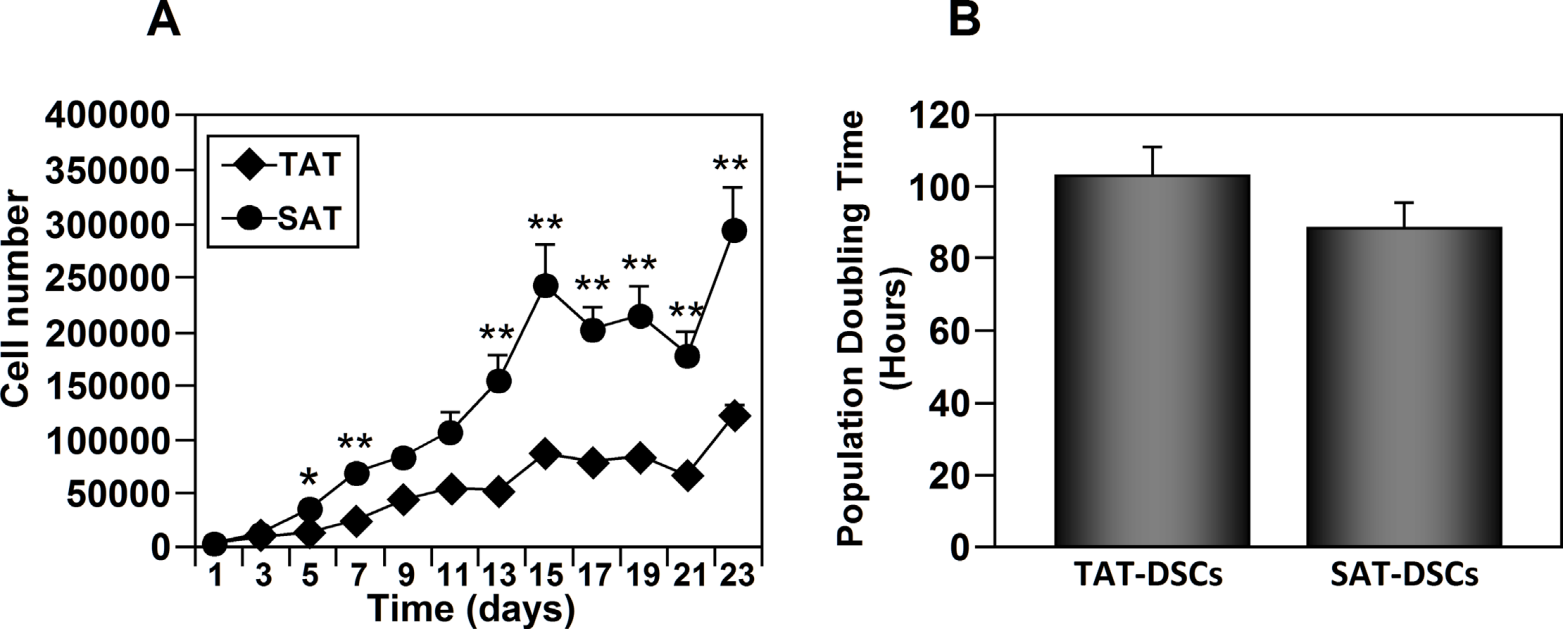
Proliferative characteristics of human thymus and subcutaneous adipose tissue derived stromal cells (TAT-ASCs and SAT-ASCs). TAT and SAT derived-stromal cells (TAT-ASCs and SAT-ASCs) were extracted from the tissues of myocardial ischemic subjects (n = 6) and were seeded in triplicate in 12 well plates at 5000 cells per cm^2^ in complete expansion medium. Cells were dissociated by trypsin and counted every 48 hours for 23 days using the Trypan Blue exclusion method. (A) Curve growth kinetics of TAT-ASCs and SAT-ASCs. Values presented in the graph represent the mean ± SE of the values obtained from each donor and which were done in triplicate. (B) The population doublings (PDs) were calculated using the following equation: PDs = 240/Log2 (N2/N1), where N2 and N1 represent the average cell number at 15th and 5th day, respectively. The comparison between the two ASC types were carried out with Mann-Whitney U-Test.*p<0.05; **p<0.01.

We next analyzed the growth rate of cultured TAT-ASCs and SAT-ASCs by counting the cell number at the indicated days (Fig 2A). The two cell types did not show significant differences in the average doubling time (103.13±8.24h for TAT-ASCs and 88.86 ±6.89h for SAT-ASCs) (Fig 2B), however, SAT-ASCs had a significantly higher proliferation rate than TAT-ASCs.

### Immunophenotype characterization of TAT-ASCs and SAT-ASCs by flow cytometry

**Fig 3:**
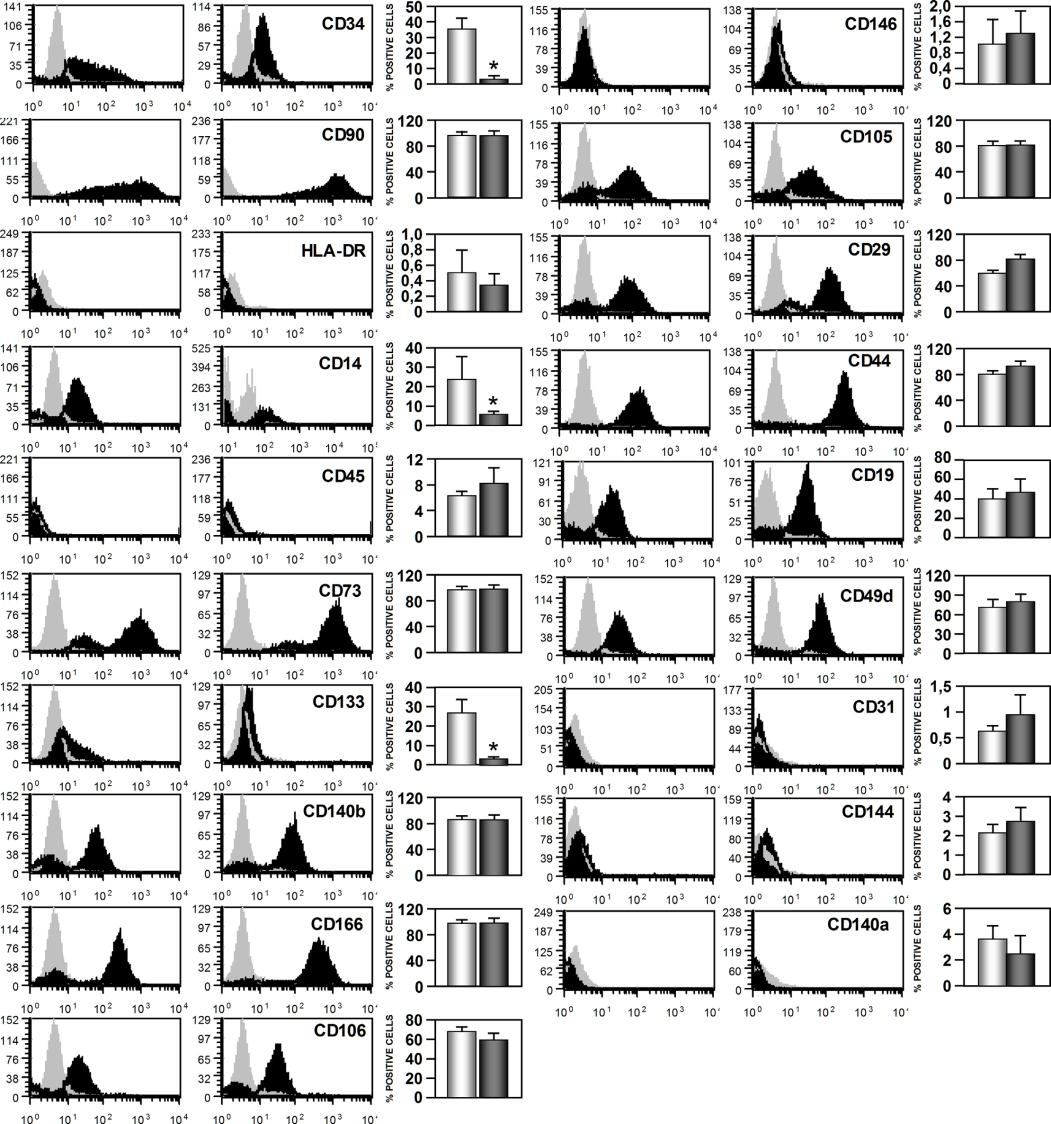
Phenotypic characterization of human thymus and subcutaneous adipose tissues derived stromal cells (TAT-ASCs and SAT-ASCs) at 3^rd^ passage. Representative histograms of six analyzed patient samples and percentage of positive cells for each of the markers analyzed of TAT-ASCs and SAT-ASCs. 1x10^5^ cells from passage 3 were labeled with the described markers or the isotype-matched control antibody. Samples were analyzed on a FACScan CyAnTM High-speed ADP Analyzer (Beckman Coulter, CA. EEUU). Values as mean ± SEM were obtained from each donor (n = 6). The comparison between the two ASC types were carried out with Mann-Whitney U-Test.*p<0.05.

We next conducted a comparative flow cytometric analysis of the identified TAT-ASCs population and SAT-ASCs in order to characterize the CD marker profiles of these cells. As shown in Fig 3, both cell types were CD105^+^, CD166^+^, CD73^+^, CD90^+^, CD29^+^, CD44^+^, CD106^+^, CD49D^+^, CD140b^+^, and negative for the hematopoietic cell surface markers CD45, HLA-DR, CD144, CD140a, CD31 and CD14; and the percentages of positive cells were similar for both cell types.

Three surface markers were distinctive between the two cell types. CD14 expression was higher in TAT-ASCs (23.81±11.76%) than in SAT-ASCs (6.00±1.12%.), and CD133 and CD34 were significantly higher in TAT-ASCs (27.06±6.82; 35.5±7.44) than in SAT-ASCs (3.29±0.50; 3.38±1.32).

### Differentiation capacity of TAT-ASCs and SAT-ASCs

**Fig 4:**
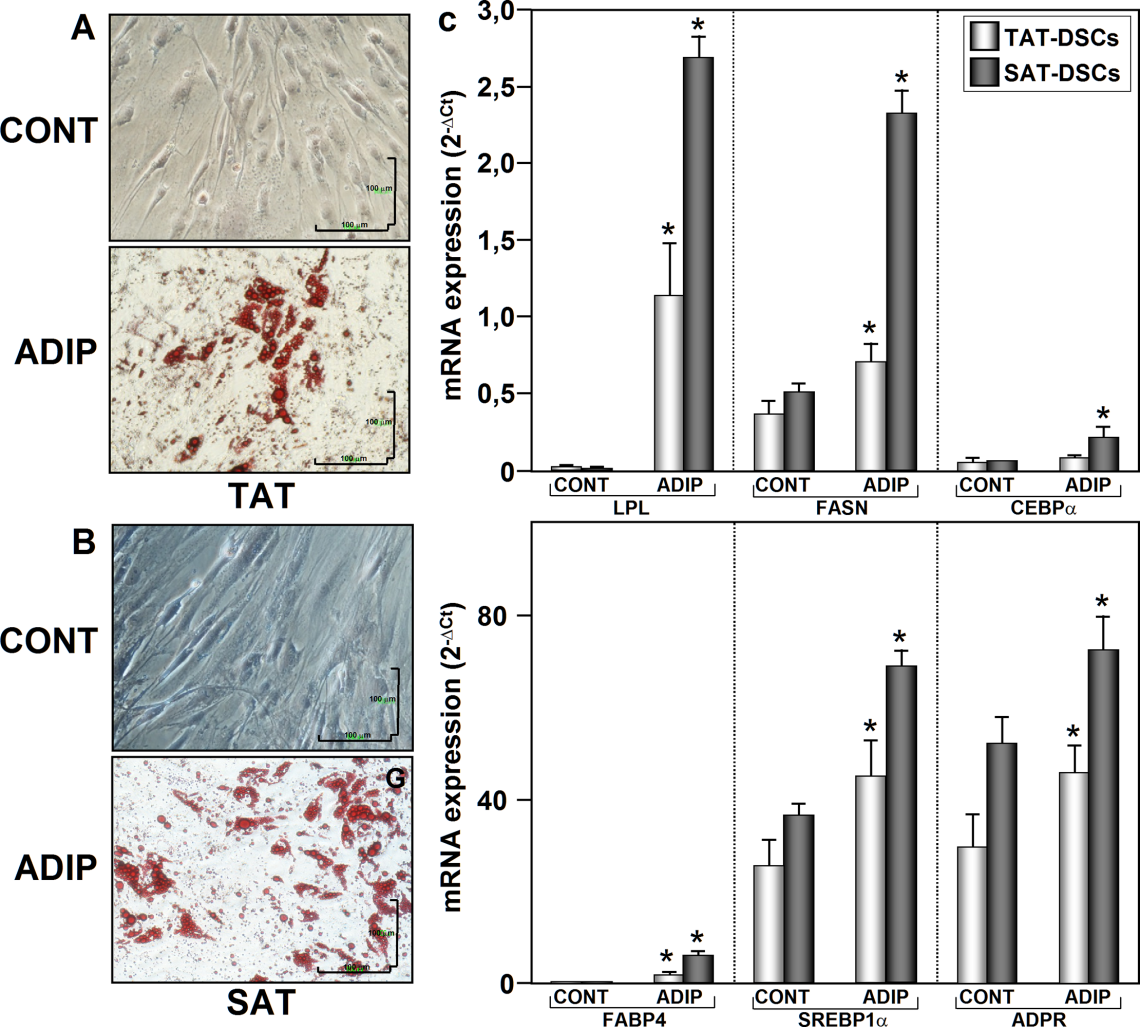
Adipogenic differentiation of human thymus and subcutaneous adipose tissues derived stromal cells (TAT-ASCs and SAT-ASCs). Representative photomicrographs of cells obtained from thymus fat (TAT-ASCs) (A) and from subcutaneous adipose tissue (SAT-ASCs) (B) (n = 26) grown in adipogenic medium for 14 days. Adipogenic differentiation of AASCs was confirmed by detecting the accumulation of the lipophilic marker, Oil Red O, over time in lipid droplets of adipose cells. (C) Adipogenic process confirmation was carried out by mRNA expression analysis of the adipogenic markers FABP4, LPL, FASN, ADRP, CEBPα and SREBP1α. mRNAs were normalized to cyclophilin expression levels. Each sample from each subject was analyzed separately and in triplicate. Data represent the mean ± SEM of the values obtained from all samples. The relation between control and adipogenic differentiation was analyzed by range test with Wilcoxon sign. ^*^p<0.05. Image magnification is 20X.

**Fig 5:**
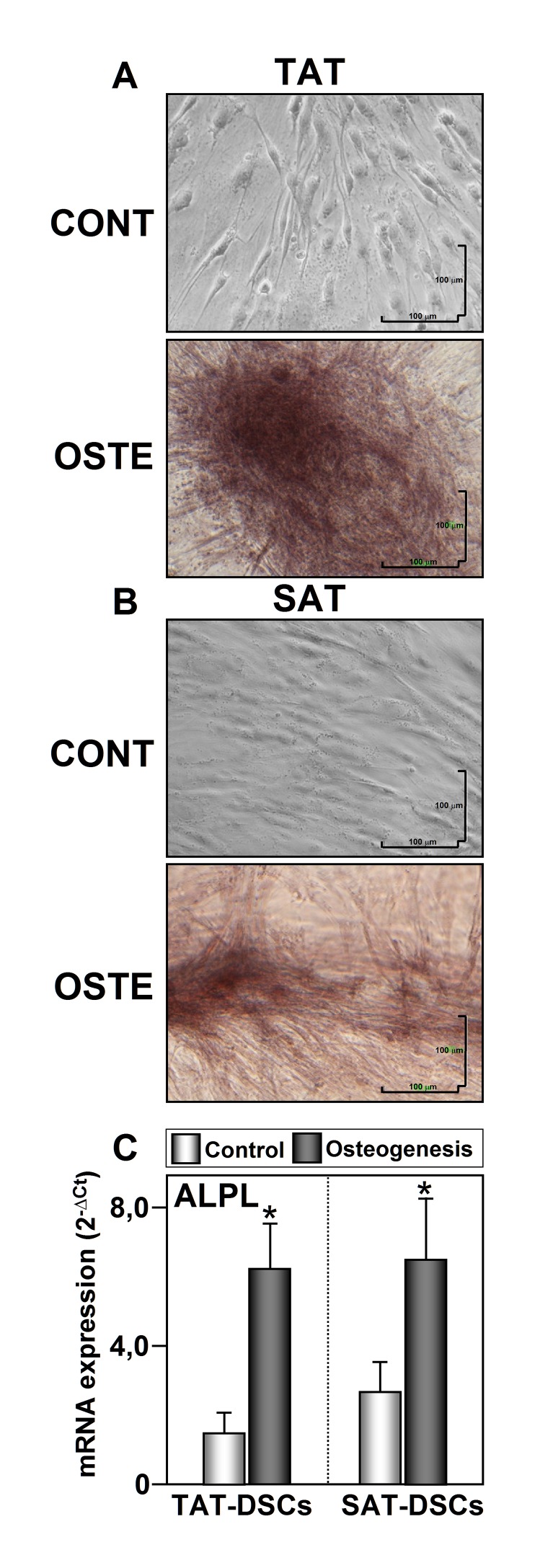
Osteogenic differentiation of human thymus and subcutaneous adipose tissues derived stromal cells (TAT-ASC and SAT-ASC). Representative photomicrographs of cells obtained from thymus fat (TAT-ASCs) (A) and from subcutaneous adipose tissue (SAT-ASCs) (B) (n = 26) grown in osteogenic medium for 14 days. Osteogenic differentiation of ASCs was confirmed by detecting calcium deposition by Alizarin Red S staining. (C) Osteogenic process confirmation was carried out by mRNA expression analysis of the osteogenic marker ALPL. mRNAs were normalized to cyclophilin expression levels. Each sample from each subject was analyzed separately and in triplicate. Data represent the mean ± SEM of the values obtained from all samples. The relationship between control and osteogenic differentiation was analyzed by range test with Wilcoxon sign. ^*^p<0.05. Image magnification is 20X.

Adipogenic and osteogenic differentiation are usually defined, respectively, by the appearance of cells containing intracellular lipid droplets under Oil Red-O staining, and enhanced alkaline phosphatase expression and mineralization under alizarin red staining. As shown in Fig 4A, both types of ASCs were able to differentiate into adipocytes after 14 days of culture in adipogenic medium as evidenced the presence of lipids droplets. In addition, increased mRNA levels of adipogenic markers were also found (Fig 4B). Enhanced alkaline phosphatase (ALPL) mRNA expression levels and positive staining by alizarin red indicated that both cell types were able to differentiate into osteoclasts in the present of osteogenic culture medium (Fig 5).

### Age-related changes in the adipogenic and osteogenic capacity of TAT-ASCs and SAT-ASCs

**Fig 6:**
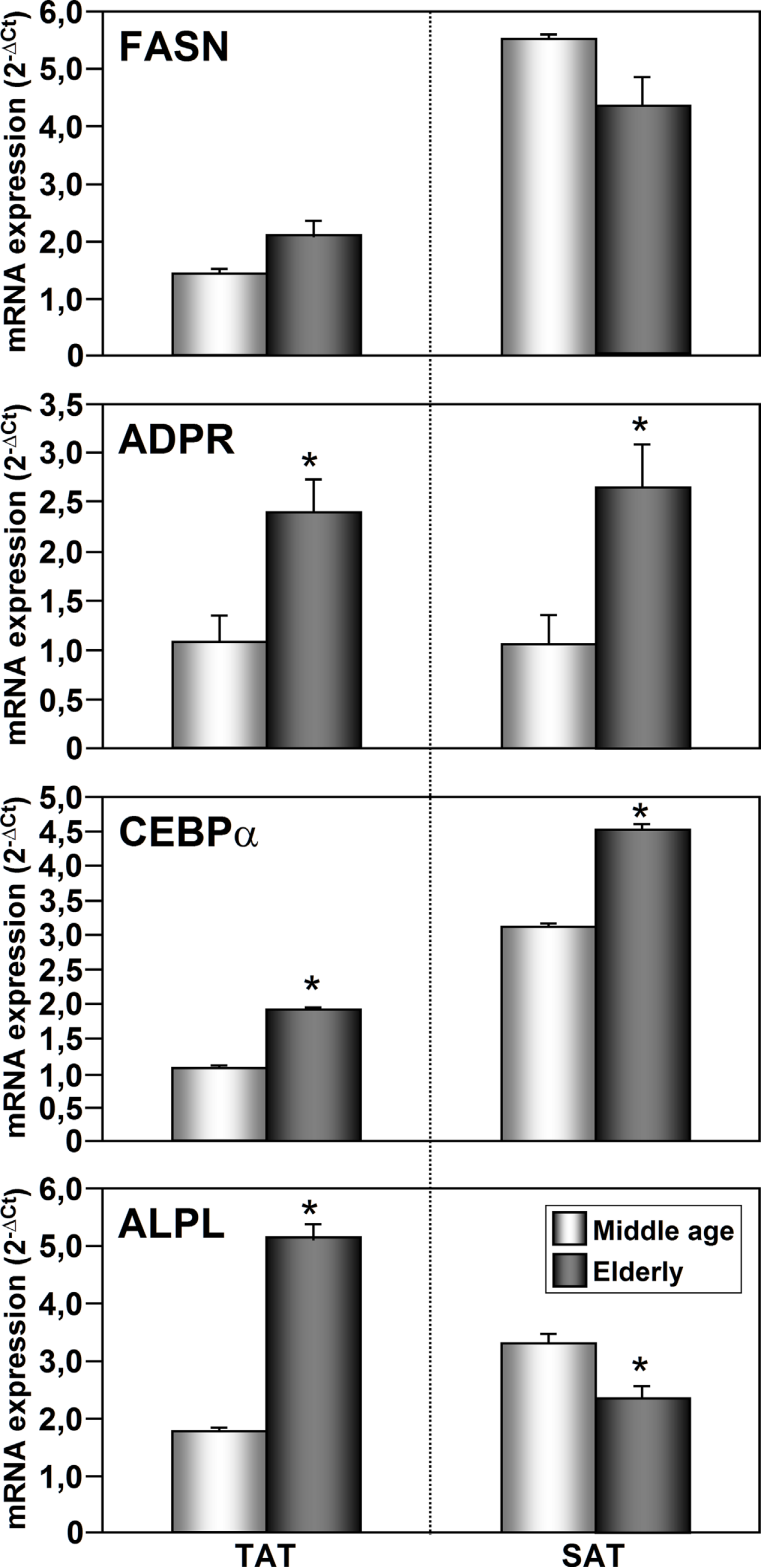
Comparative analysis of adipogenic and osteogenic marker mRNA expression levels between differentiated ASCs from elderly and middle aged myocardial ischemic subjects. FASN, ADRP, CEBPα and ALPL mRNA expression levels were analyzed in human adult thymus fat and subcutaneous adipose tissues (TAT-ASCs and SAT-ASCs) from middle age (45–65 years, n = 8) and elderly (> 70 years, n = 18) subjects. Each sample from each subject was analyzed separately and in triplicate. Data represent the mean ± SEM of the values obtained from all samples. The comparison between the two groups were carried out with Mann-Whitney U-Test.*p<0.05.

In order to analyze whether the age could influence the differentiation capacity of TAT-ASCs and SAT-ASCs, we next analyzed the expression levels of adipogenic and osteogenic markers expression levels according to the age of the subjects. As shown in Fig 6, adipogenic differentiated TAT-ASCs and SAT-ASCs from elderly subjects showed higher levels of the adipogenic markers ADRP and CEBPα mRNAs than those from middle-aged subjects. TAT-ASCs from elderly subjects showed higher levels of the osteogenic marker ALPL than those from middle-aged subjects, while SAT-ASCs from elderly subjects showed lower levels of this marker compared with cells from the middle-aged group (Fig 6).

## Discussion

Adipose Tissue–Derived Stromal Cells (AT-ASCs) are one of the most promising sources of stem cells for tissue engineering applications [14,15]. Several groups have shown that stem cells from the SVF of SAT display multi-lineage developmental plasticity [8,16]. It is therefore important to identify and characterize new sources of these cells within ATs in order to select the best suited for such applications. The aim of this study was to investigate the easily accessible thymus fat from adult subjects with myocardial ischemia as a source of AT-ASCs in comparison with SAT-ASCs.

The results described here show that the human adult thymus fat is a good candidate source of ASCs. Several lines of evidence verify this claim: 1) TAT-ASCs present fibroblastic morphology and are able to adhere to plastic; 2) They can be cultured and expanded over 20 cycles without showing signs of senescence [3,17]; 3) They have the ability to form CFU-F; 4) They have the capacity to differentiate into osteocytes and adipocytes lineages; and 5) They present a surface marker profile corresponding with stem cells derived from AT; CD73+CD90+CD105+CD14-CD19-CD45-HLA-DR- [4,18,19]. As shown, these parameters were similar between TAT-ASCs and SAT-ASCs. All together these data indicate that TAT-ASCs meet the criteria established by the International Society for Cellular Therapy (ISCT) [8]. In our analysis we found that SAT-ASCs have the tendency to have lower average doubling time compared to those from TAT-ASCs, however, the differences are not significant. These subtle differences seem to be enough to make a cumulative effect that is shown as a significant increase of the total cell number since day 7 of proliferation.

On the other hand, TAT-ASCs showed high capacity to differentiate into the most relevant cells lineages, osteocytes and adipocytes.

In opposition to SAT-ASCs and other AT-ASCs [3,20], the immunophenotype analysis showed the presence of CD34^+^ CD133^+^ and CD45^-^ cells which differs from those detected in SAT-ASCs, this suggesting the potential existence of a subpopulation CD34^+^ CD133^+^ CD45^-^, which has been described as endothelial progenitor cells that contribute to vasculogenic activity and vessels formation [21,22]. It is important to highlight that CD34 is a known marker of hematopoietic stem cells, vascular endothelial cells and their precursors [23–25]. Here CD45^-^ phenotype of TAT-ASCs precludes contamination by haematopoietic cells [26]. In agreement with our data, Russo *et al*. have recently described that ASCs isolated from thymus remnant fat had a longer average doubling time and a significantly higher proportion of CD34^+^ cells, as compared with cells isolated from subcutaneous fat or the omentum [11].

CD14^+^ cells have been previously described as cells with the capacity to differentiate into functional endothelial cells and so-called “endothelial-like cells” or ‘early endothelial progenitor cells" (EPC) [27–29]. Here we show that in stark contrast to other known AT-ASCs [32], TAT-ASCs contain CD14^+^ cells. Interestingly, and also in opposition to what happens specifically in SAT-ASCs, TAT-ASCs contain both CD14^+^ and CD34^+^ cells. Altogether the discovery of the CD34^+^CD133^+^CD14^+^ subpopulation from TAT-ASCs have great interest for the vascular regenerative medicine scientific community, as it has been shown that interaction of CD14^+^ cells with CD34^+^ cells potentiates the capacity of CD14^+^ cells to differentiate into endothelial-like cells [30–33]. Here we show that both ASCs are CD146^-^ cells. The expression of this cellular marker is well known to decrease with successive culture passages [34].

One important feature of stem cells is the differentiation capacity. It has been previously described that SAT-ASCs have higher differentiation capacity than other AT-ASCs [35–37]. In this sense, we found that the differentiation capacity of TAT-ASCs is similar to that of SAT- ASCs. This suggests that TAT-ASCs could represent another suitable source of ASCs.

It has been described that aging declines the proliferation rate, the doubling time as well as the differentiation capacities of AT-ASCs [38,39]. The differentiation capacity is widely accepted to be associated with a decreased expression of key regulator of adipogenic events [40–42]. On the other hand, we found that aging increased the differentiation capacity of SAT-ASCs and TAT-ASCs from patients with myocardial ischemia rather than decrease it, as shown by the increased levels of the adipogenic markers, ADRP and CEBPα, in cells from elderly subjects (Fig 6).

The osteogenic differentiation capacity of AT-ASCs has been described to get progressively impaired with aging [38,43,44]. Here we show that TAT-ASCs and SAT-ASCs have similar osteogenic differentiation capacity. However, this capacity was higher in TAT-ASCs than in SAT-ASCs with age as evidenced by the higher expression of the master oteogenic regulator, ALPL, in TAT-ASCs from elderly subjects than from middle age subjects. Our results highlight that it is of interest to evaluate the potential use of TAT-ASCs in osteogenic regeneration and therapy in elderly subjects.

It is well known that osteoblasts and adipocytes share a common mesenchymal ancestor [45]. Recent *in vitro* evidence suggests that agonists of osteogenic differentiation act as antagonists of adipogenic diferentiation and *vice versa* [46]. Our results highlight that adipogenic differentiation capacity was increased in both aging TAT and SAT, while osteogenic differentiation capacity was decreased in SAT but significantly increased in TAT. This raises the questions of whether the inverse relationship between adipogenic and osteogenic capacities, is applicable to TAT from myocardial ischemic subjects. Further research is required to illuminate this important matter.

In summary, in spite of the fact that ASCs are an appealing source of cells for therapeutic intervention, the environment from which these cells are isolated may impact their usefulness. To date, the age, the adipose tissue depot site, and some pathologies such as metabolic syndrome have been shown to affect negatively stemness and angiogenic capacity of ASCs [47–49], suggesting that caution should be exercised when considering the source of ASCs for cellular therapies since their therapeutic potential may be impaired [50]. Another source of stem cells that is commonly investigated is bone marrow, however, it is widely accepted that aging impairs their proliferation and differentiation potentials. In fact, it has been shown that aging decreases proliferation capacity, senescence and doubling time of bone marrow derived stem cells compared to adipose tissue derived stem cells [51,52]. These data evidence that in elderly subjects the AT is a more suitable source of stem cells than the bone marrow.

Altogether, our data suggest that TAT from elderly cardiomyopathy ischemic subjects may be a suitable source of ASCs for the cell-based neovascularization therapy in general, and for subjects with myocardial ischemia, in particular.

## Supporting Information

S1 Fig Complete set of the original data presented in Fig 3. (PDF)

S1 Table Antibodies used in this study. FITC: fluorescein isothiocyanate. PE: phycoerythrin. PE-Cy^TM^7: tandem fluorochrome that combines phycoerythrin and cyanine dye. APC: allophycocyanin.
(DOC)

## Abbreviations

FABP4: Fatty Acid Binding Protein 4
ADRP: Adipocyte Differentiation-Related Protein
CEBPα: CCAAT-enhancer-binding protein alpha
ALP: alkaline phosphatase
ASCs: Adipose tissue- Stromal Cells
TAT: Thymus Adipose Tissue
SAT: Subcutaneous Adipose Tissue
PDs: The population doublings
CFU-F: Colony Forming Unit-Fibroblastic
