# Supplementary material for: Myocardial Ischemic Subject’s Thymus Fat: A Novel Source of Multipotent Stromal Cells
Source: PLoS One. 2015 Dec 10;10(12):e0144401. doi: 10.1371/journal.pone.0144401 (PMC4675557; doi:10.1371/journal.pone.0144401)
Supplement: S1 Fig — (PDF) [file pone.0144401.s001.pdf]

SUBJECT 1: TAT-ASCs

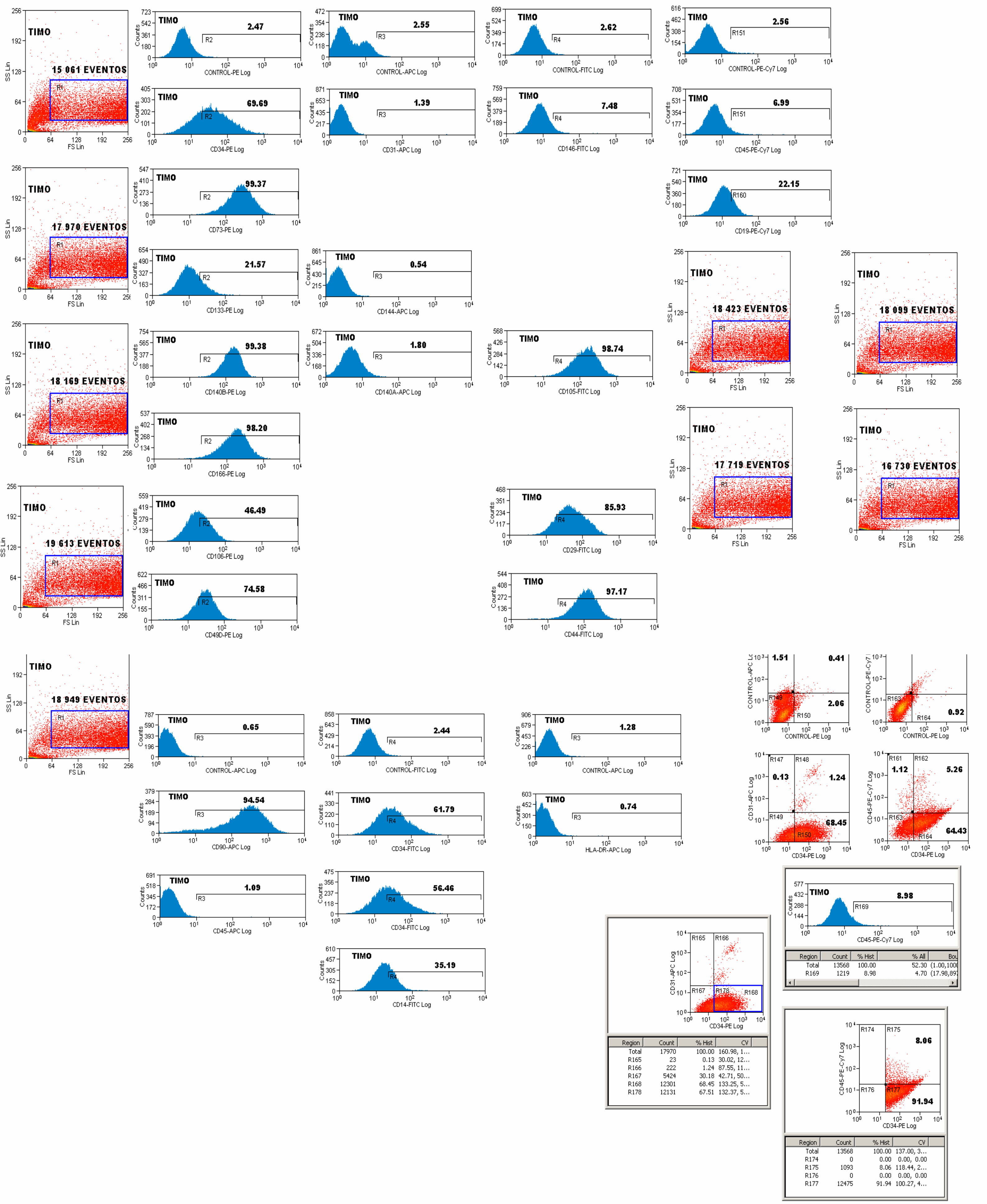

Region	Count	% Hist	CV
Total	17970	100.00	160.98, 1...
R165	23	0.13	30.02, 12...
R166	222	1.24	87.55, 11...
R167	5424	30.18	42.71, 50...
R168	12301	68.45	133.25, 5...
R178	12131	67.51	132.37, 5...

Region	Count	% Hist	% All	Bou
Total	13568	100.00	52.30	(1.00,100
R169	1219	8.98	4.70	(17.98,89

Region	Count	% Hist	CV
Total	13568	100.00	137.00, 3...
R174	0	0.00	0.00, 0.00
R175	1093	8.06	118.44, 2...
R176	0	0.00	0.00, 0.00
R177	12475	91.94	100.27, 4...

SUBJECT 1: SAT-ASCs

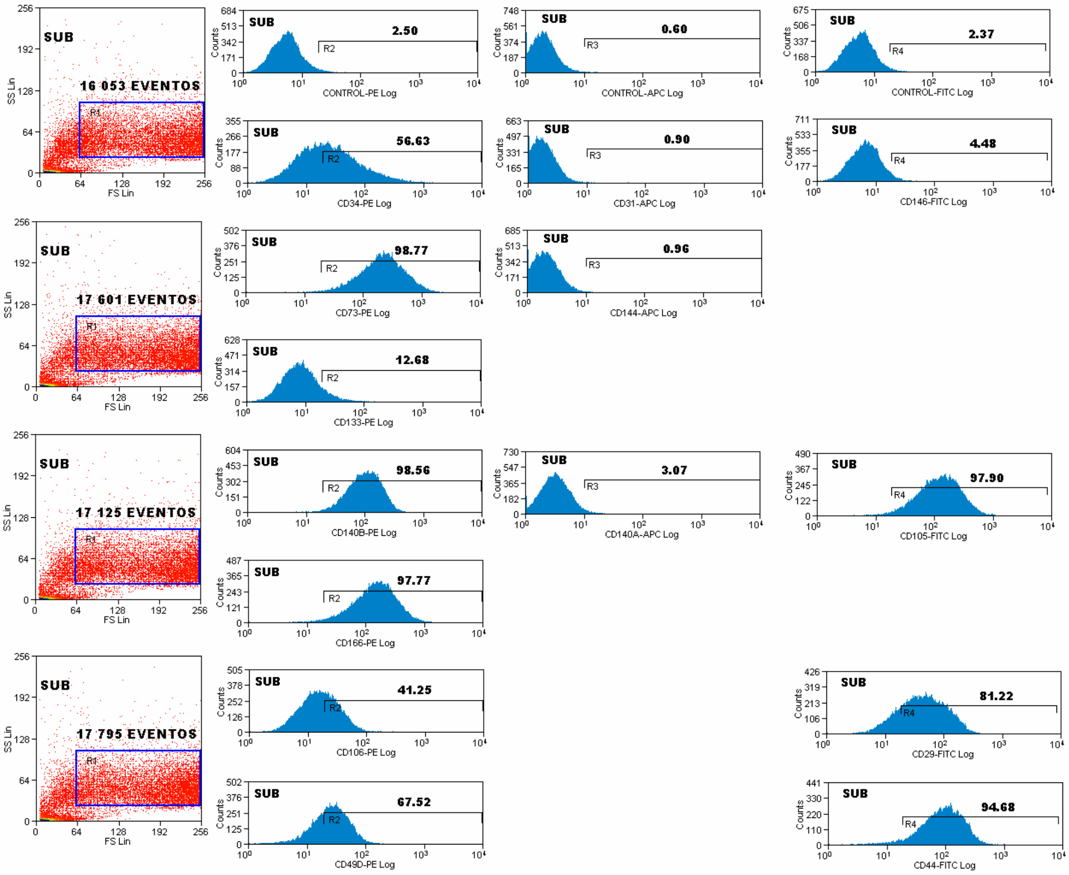

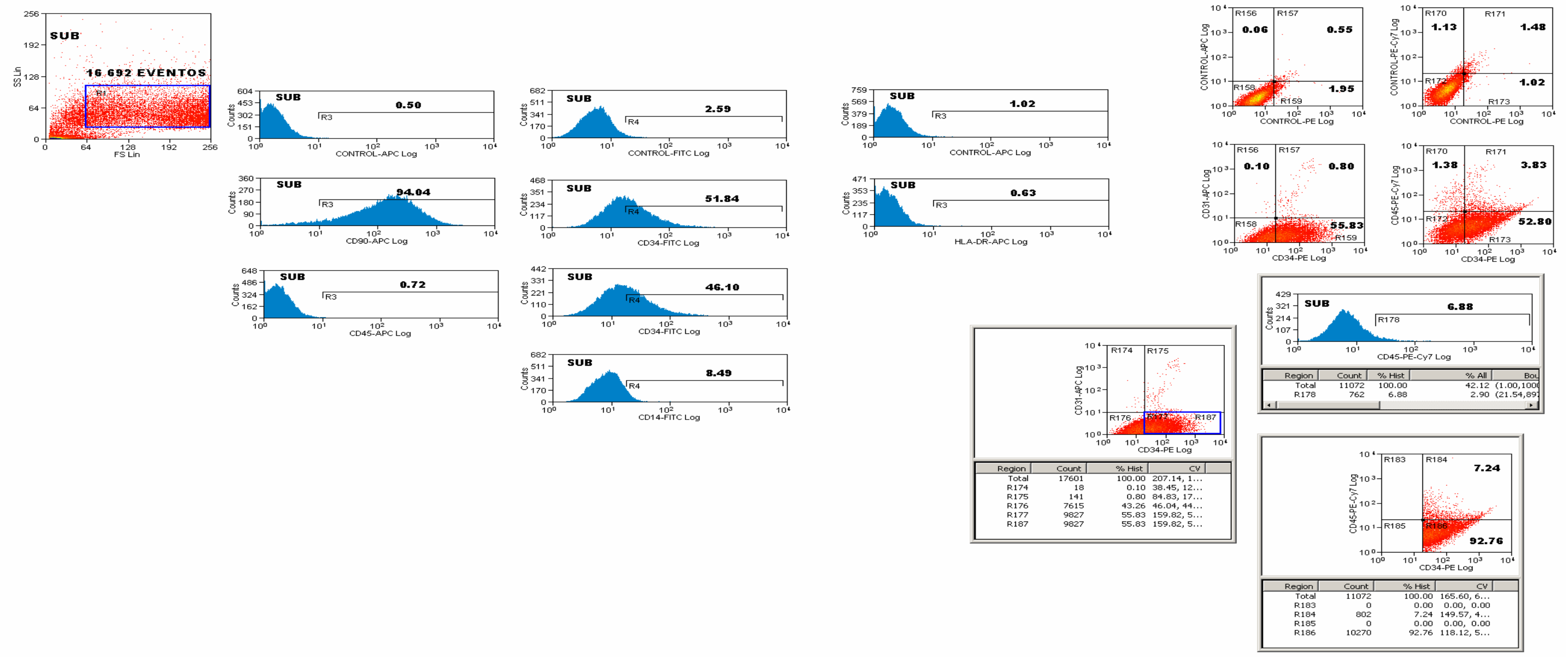

Region	Count	% Hist	CV
Total	17601	100.00	207.14, 1...
R174	18	0.10	38.45, 12...
R175	141	0.80	84.83, 17...
R176	7615	43.26	46.04, 44...
R177	9827	55.83	159.82, 5...
R187	9827	55.83	159.82, 5...

Region	Count	% Hist	% All	Bou
Total	11072	100.00	42.12	(1.00,100(
R178	762	6.88	2.90	(21.54,89

Region	Count	% Hist	CV
Total	11072	100.00	165.60, 6...
R183	0	0.00	0.00, 0.00
R184	802	7.24	149.57, 4...
R185	0	0.00	0.00, 0.00
R186	10270	92.76	118.12, 5...

SUBJECT 2: TAT-ASCs

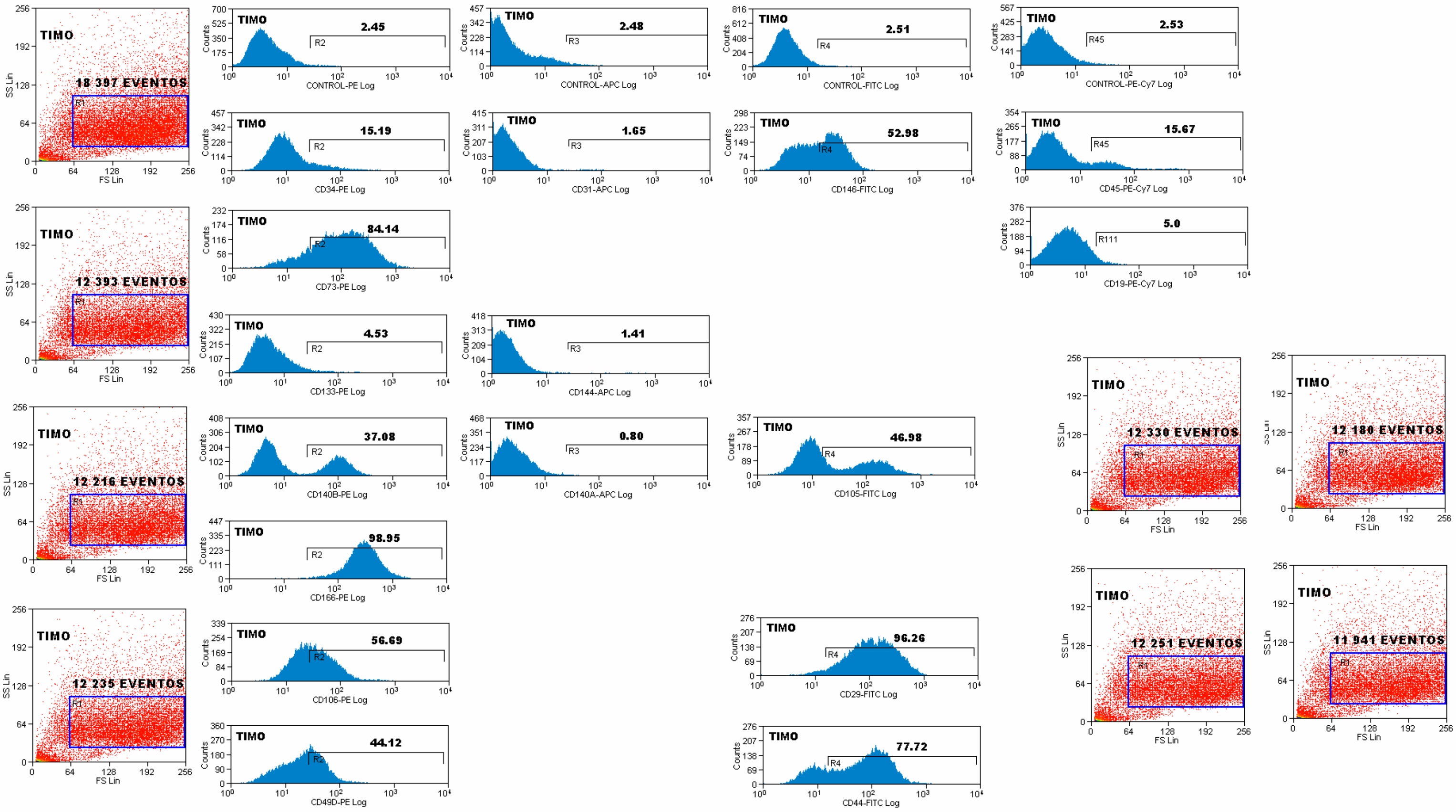

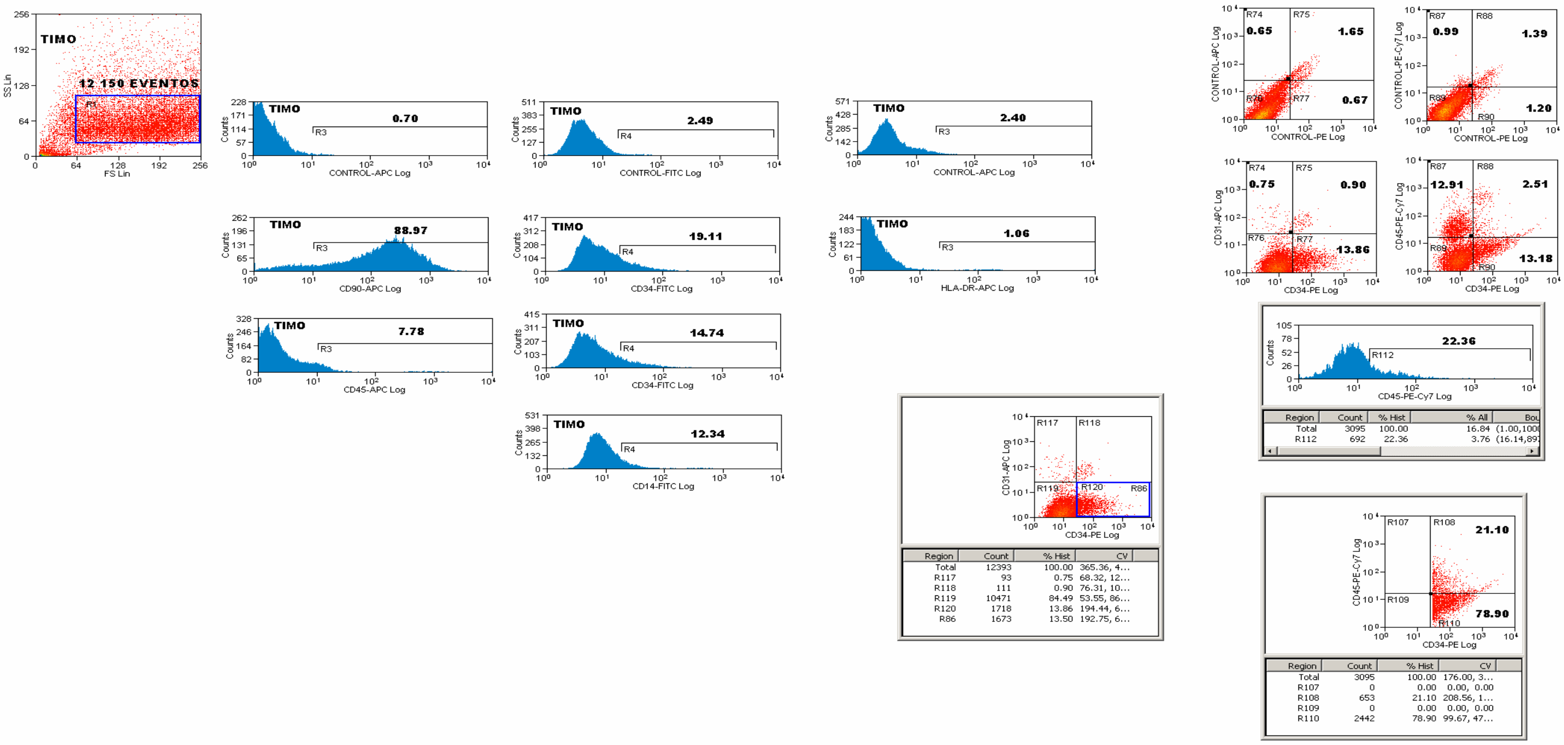

Region	Count	% Hist	CV
Total	12393	100.00	365.36, 4...
R117	93	0.75	68.32, 12...
R118	111	0.90	76.31, 10...
R119	10471	84.49	53.55, 86...
R120	1718	13.86	194.44, 6...
R86	1673	13.50	192.75, 6...

Region	Count	% Hist	% All	Bou
Total	3095	100.00	16.84	(1.00,100[illegible]
R112	692	22.36	3.76	(16.14,89[illegible]

Region	Count	% Hist	CV
Total	3095	100.00	176.00, 3...
R107	0	0.00	0.00, 0.00
R108	653	21.10	208.56, 1...
R109	0	0.00	0.00, 0.00
R110	2442	78.90	99.67, 47...

SUBJECT 2: SAT-ASCs

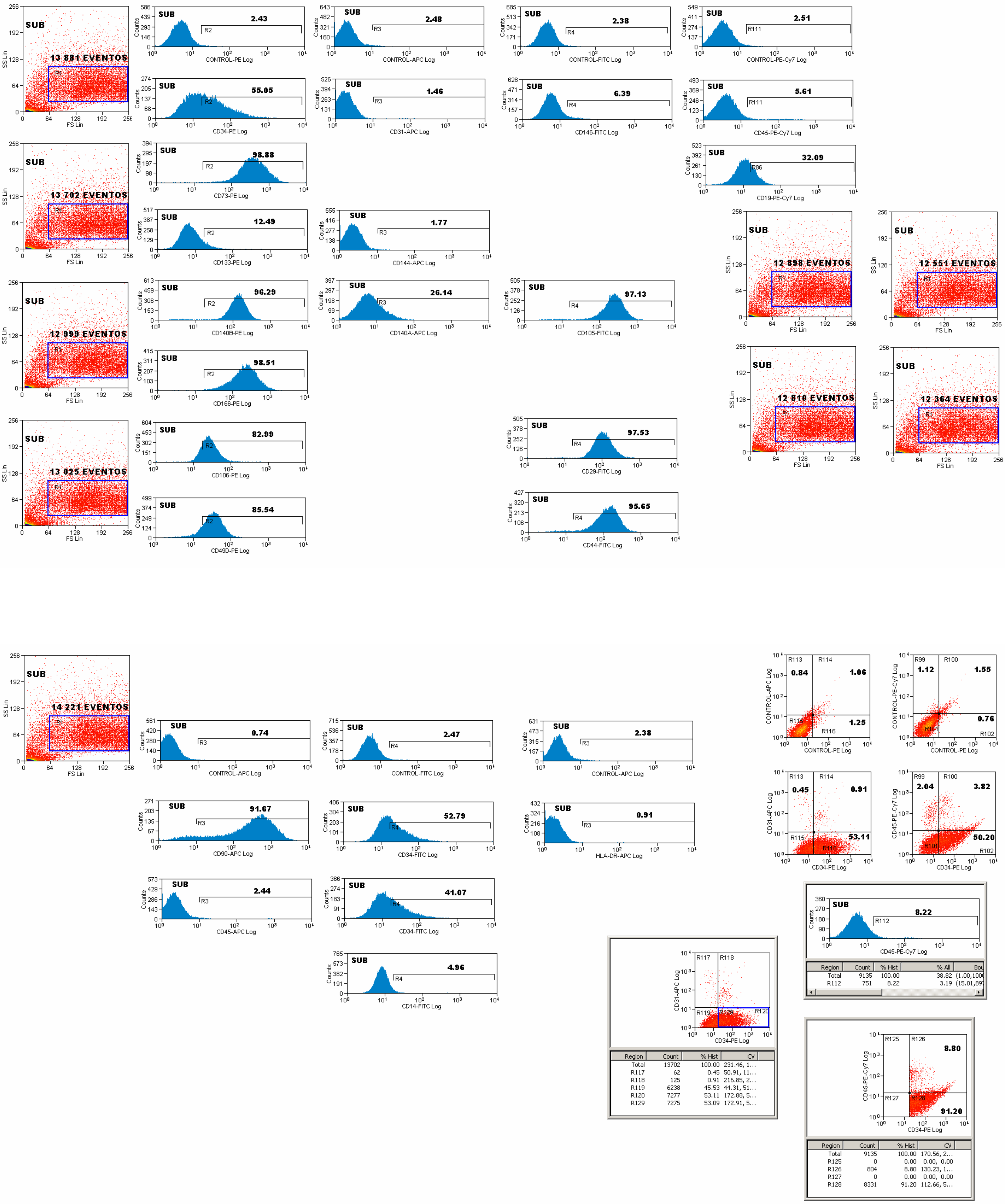

Region	Count	% Hist	CV
Total	13702	100.00	231.46, 1…
R117	62	0.45	50.91, 11…
R118	125	0.91	216.85, 2…
R119	6238	45.53	44.31, 51…
R120	7277	53.11	172.88, 5…
R129	7275	53.09	172.91, 5…

Region	Count	% Hist	% All	Bou
Total	9135	100.00	38.82	(1.00,1000
R112	751	8.22	3.19	(15.01,897

Region	Count	% Hist	CV
Total	9135	100.00	170.56, 2…
R125	0	0.00	0.00, 0.00
R126	804	8.80	130.23, 1…
R127	0	0.00	0.00, 0.00
R128	8331	91.20	112.66, 5…

SUBJECT 3: TAT-ASCs

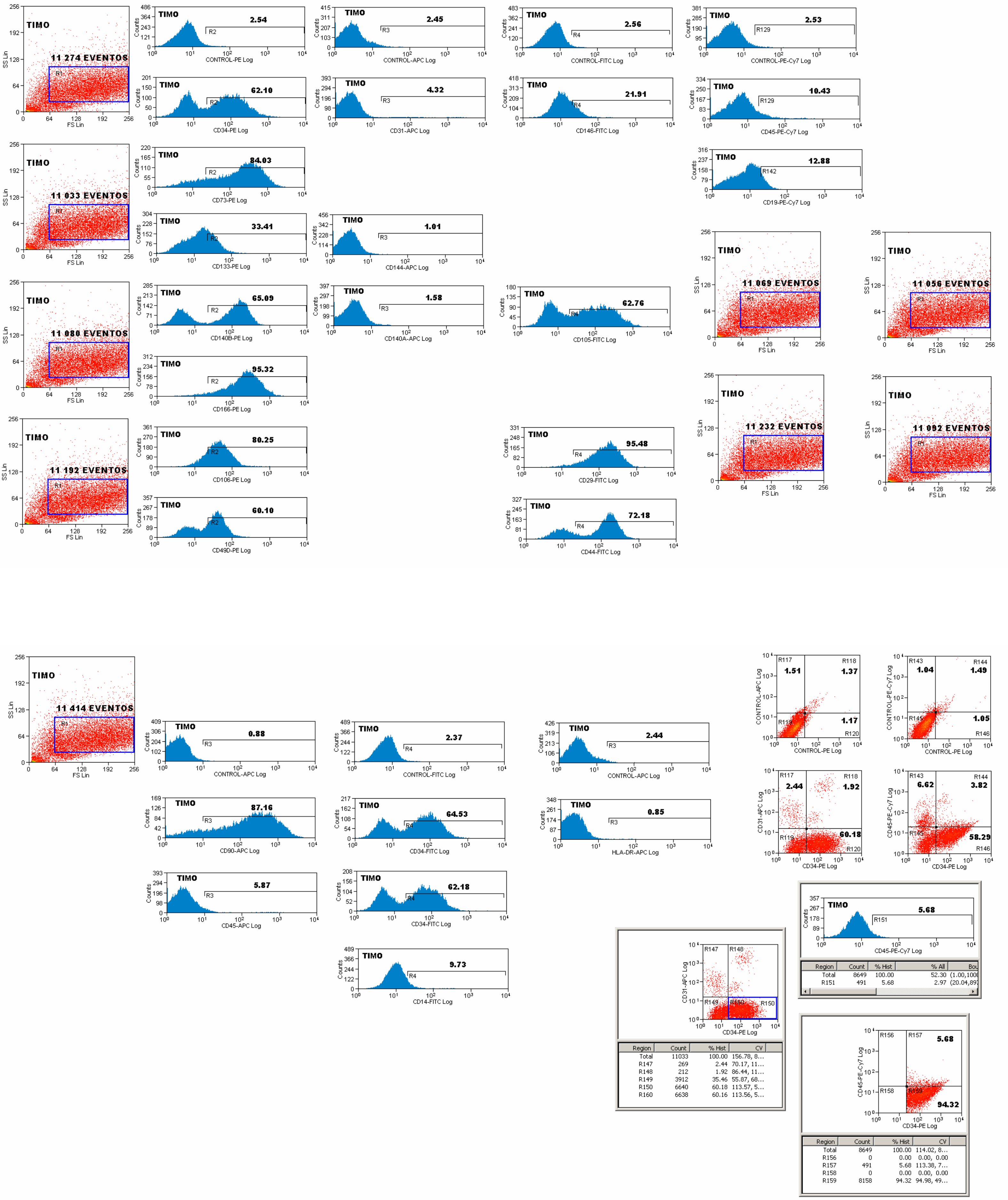

Region	Count	% Hist	CV
Total	11033	100.00	156.78, 8...
R147	269	2.44	70.17, 11...
R148	212	1.92	86.44, 11...
R149	3912	35.46	55.87, 68...
R150	6640	60.18	113.57, 5...
R160	6638	60.16	113.56, 5...

Region	Count	% Hist	% All	Bou
Total	8649	100.00	52.30	(1.00,100
R151	491	5.68	2.97	(20.04,89

Region	Count	% Hist	CV
Total	8649	100.00	114.02, 8...
R156	0	0.00	0.00, 0.00
R157	491	5.68	113.38, 7...
R158	0	0.00	0.00, 0.00
R159	8158	94.32	94.98, 49...

SUBJECT 3: SAT-ASCs

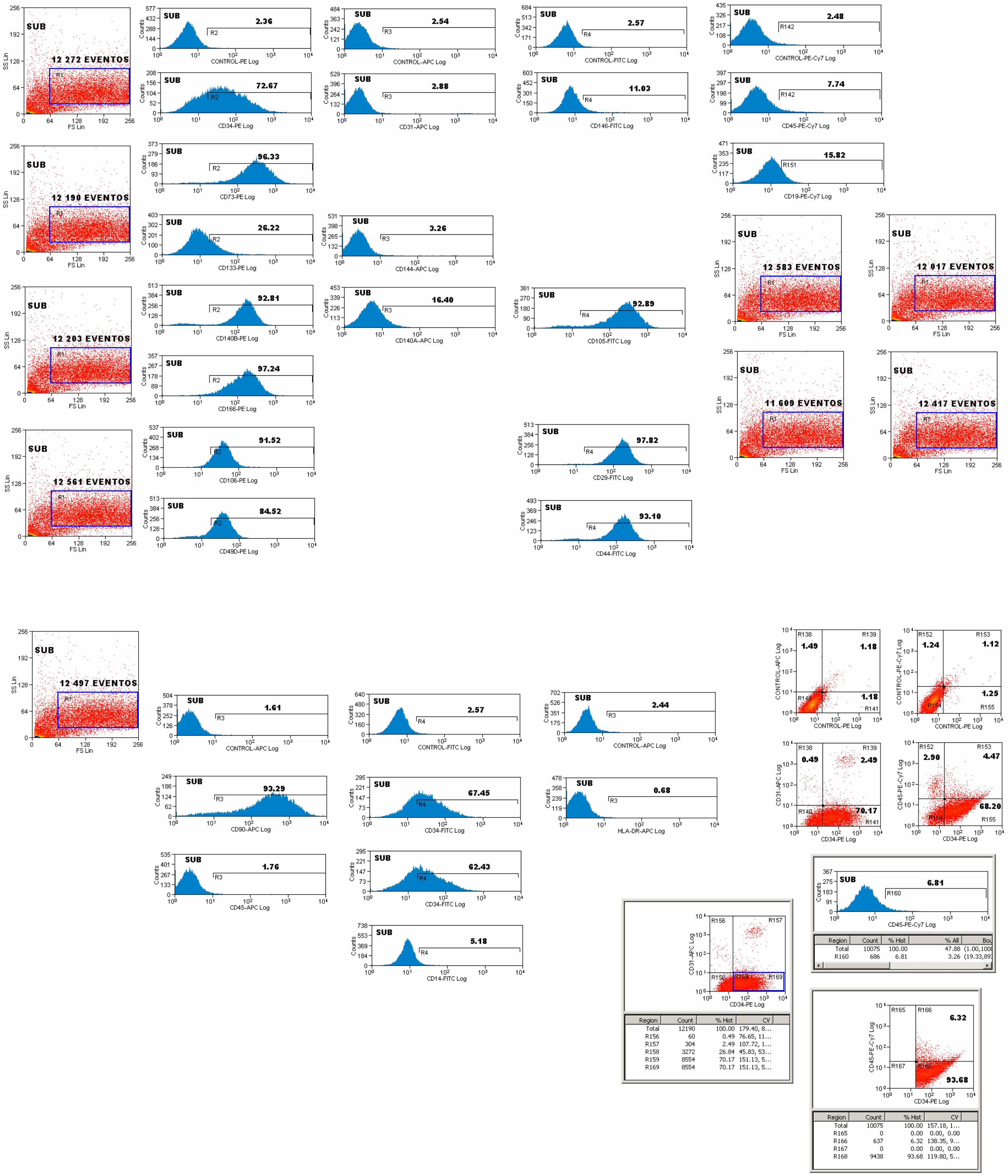

SUBJECT 4: TAT-ASCs

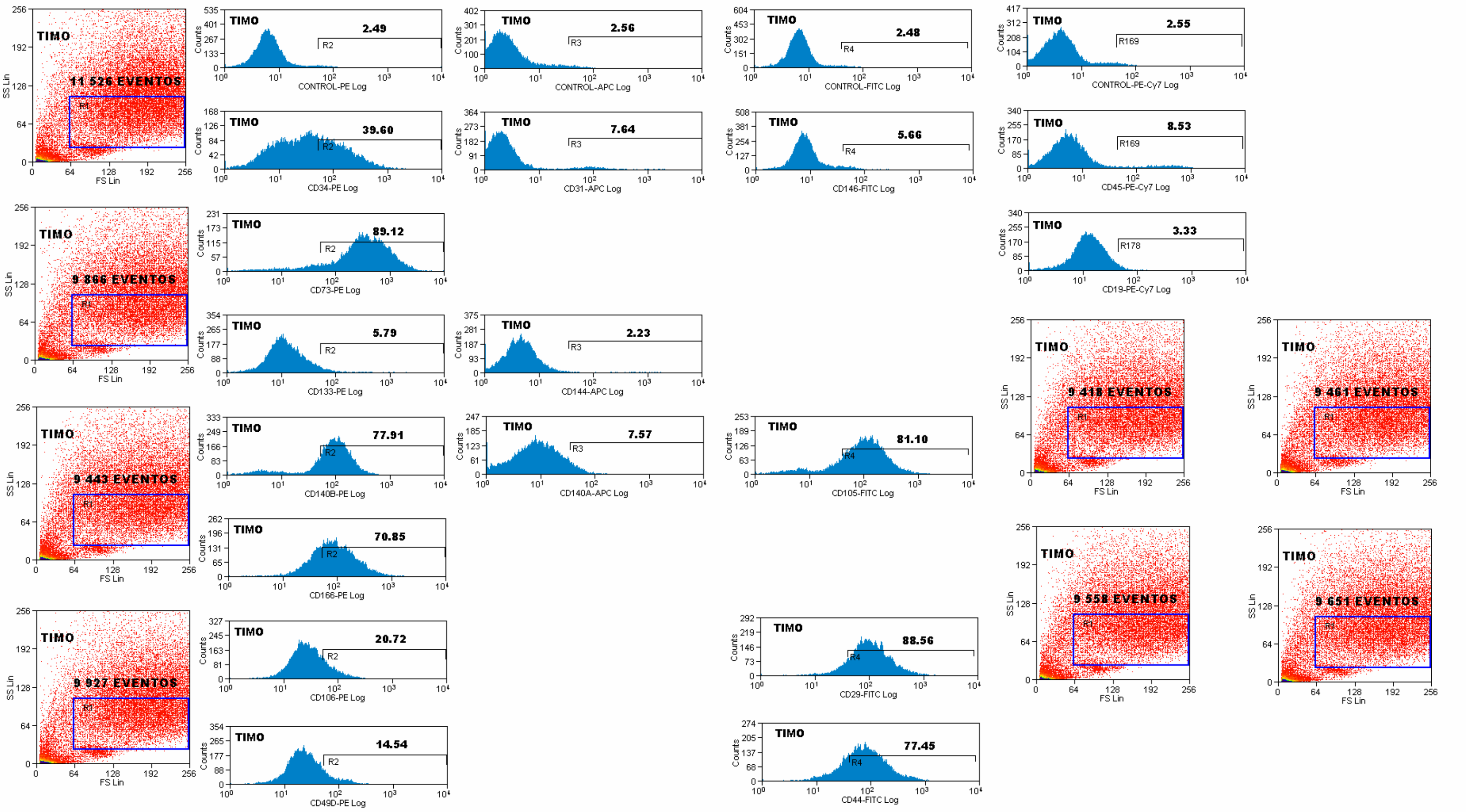

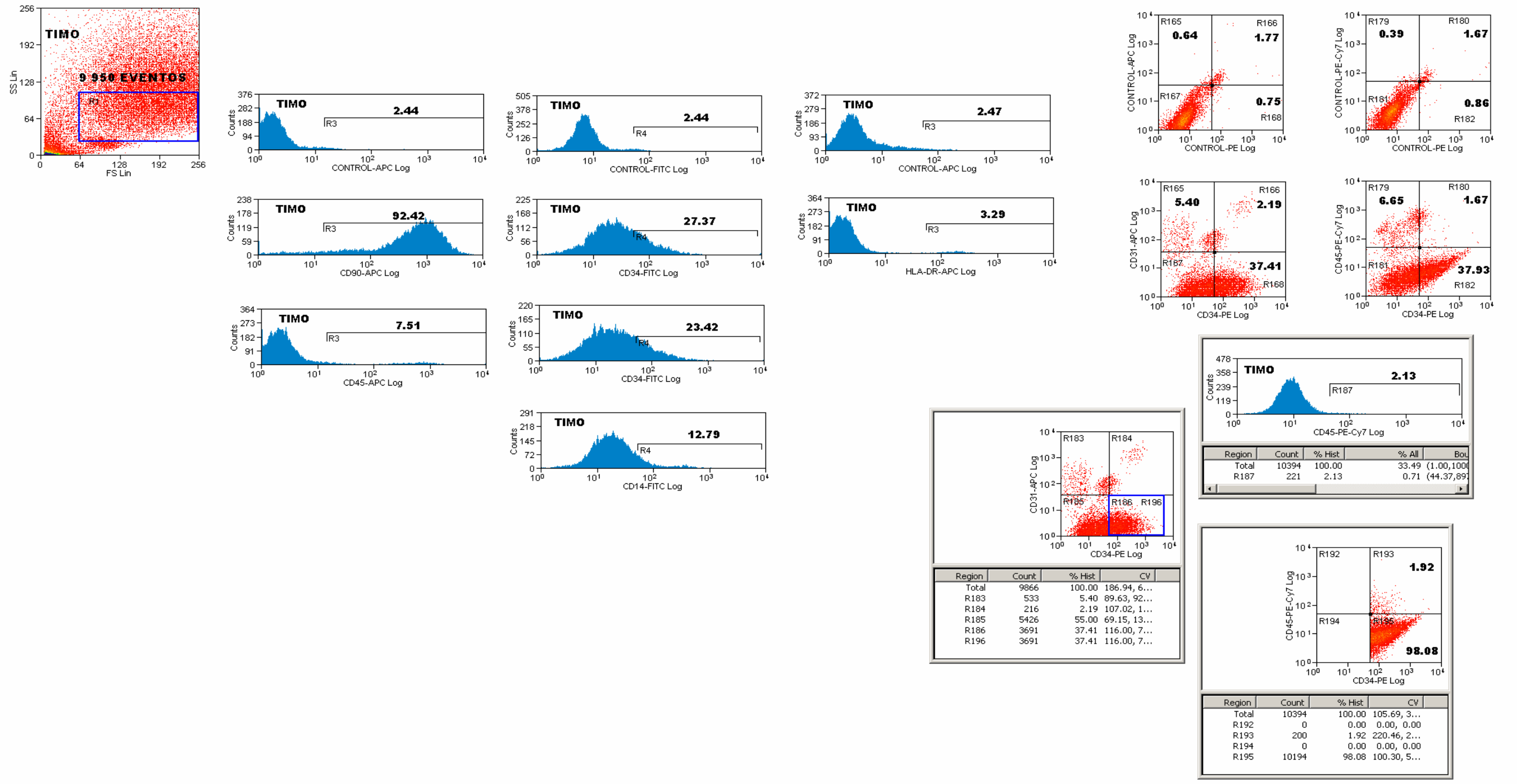

Region	Count	% Hist	CV
Total	9866	100.00	186.94, 6...
R183	533	5.40	89.63, 92...
R184	216	2.19	107.02, 1...
R185	5426	55.00	69.15, 13...
R186	3691	37.41	116.00, 7...
R196	3691	37.41	116.00, 7...

Region	Count	% Hist	% All	Bou
Total	10394	100.00	33.49	(1.00,1000
R187	221	2.13	0.71	(44.37,897

Region	Count	% Hist	CV
Total	10394	100.00	105.69, 3...
R192	0	0.00	0.00, 0.00
R193	200	1.92	220.46, 2...
R194	0	0.00	0.00, 0.00
R195	10194	98.08	100.30, 5...

SUBJECT 4: SAT-ASCs

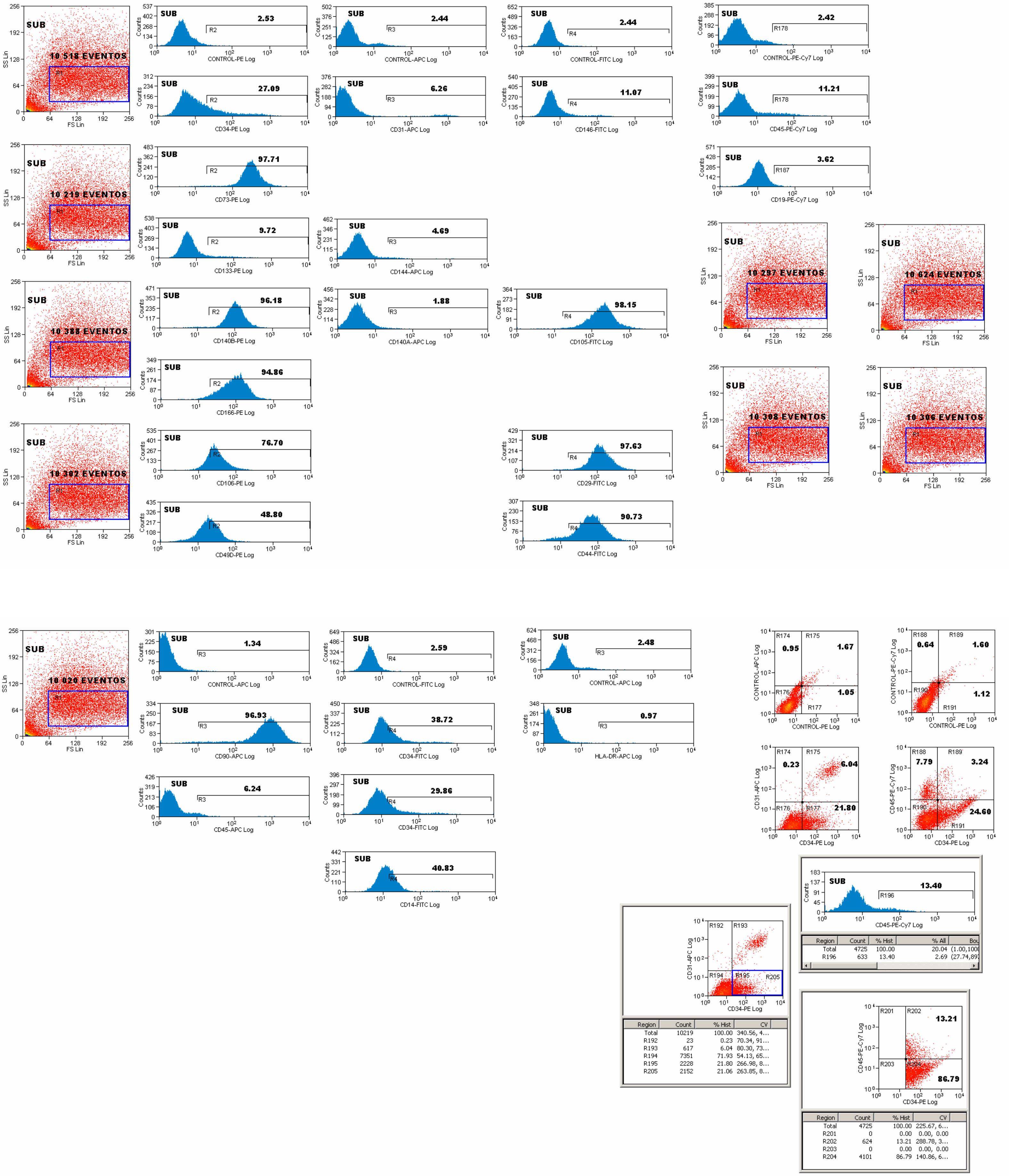

Region	Count	% Hist	% All	Bou
Total	4725	100.00	20.04	(1.00,100
R196	633	13.40	2.69	(27.74,89

Region	Count	% Hist	CV
Total	10219	100.00	340.56, 4...
R192	23	0.23	70.34, 91...
R193	617	6.04	80.30, 73...
R194	7351	71.93	54.13, 65...
R195	2228	21.80	266.98, 8...
R205	2152	21.06	263.85, 8...

Region	Count	% Hist	CV
Total	4725	100.00	225.67, 6...
R201	0	0.00	0.00, 0.00
R202	624	13.21	288.78, 3...
R203	0	0.00	0.00, 0.00
R204	4101	86.79	140.86, 6...

SUBJECT5: TAT-ASCs

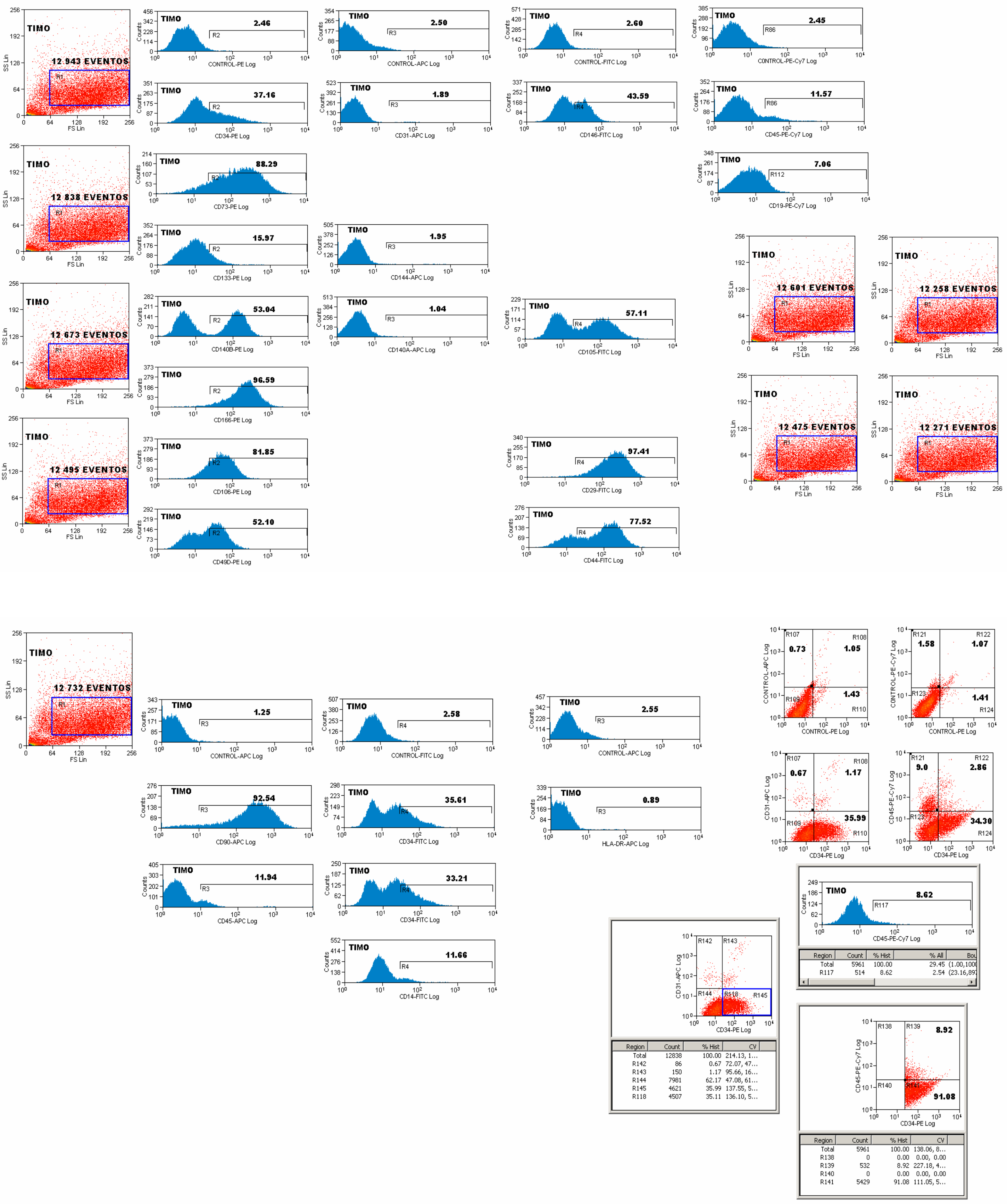

SUBJECT5: SAT-ASCs

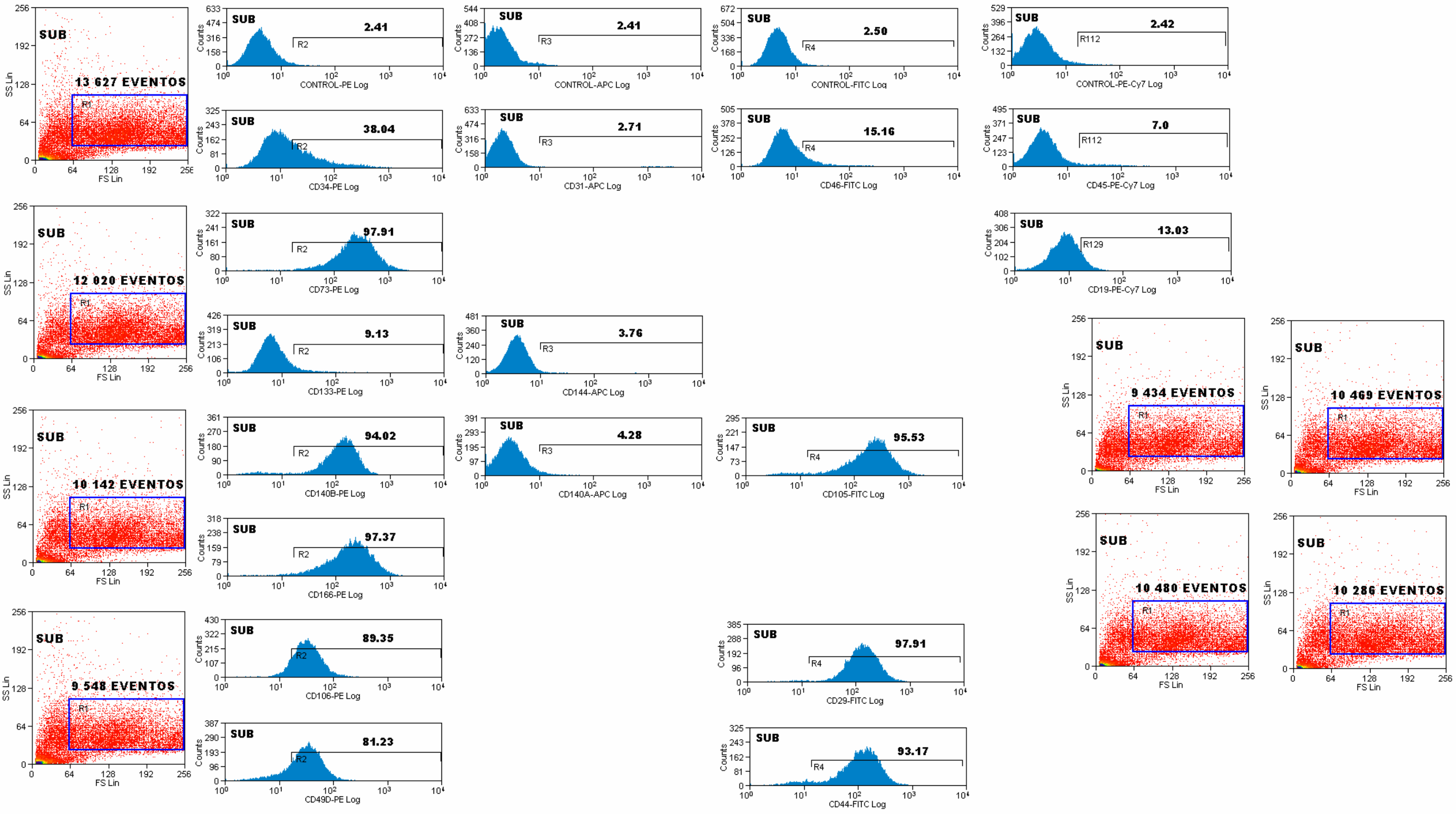

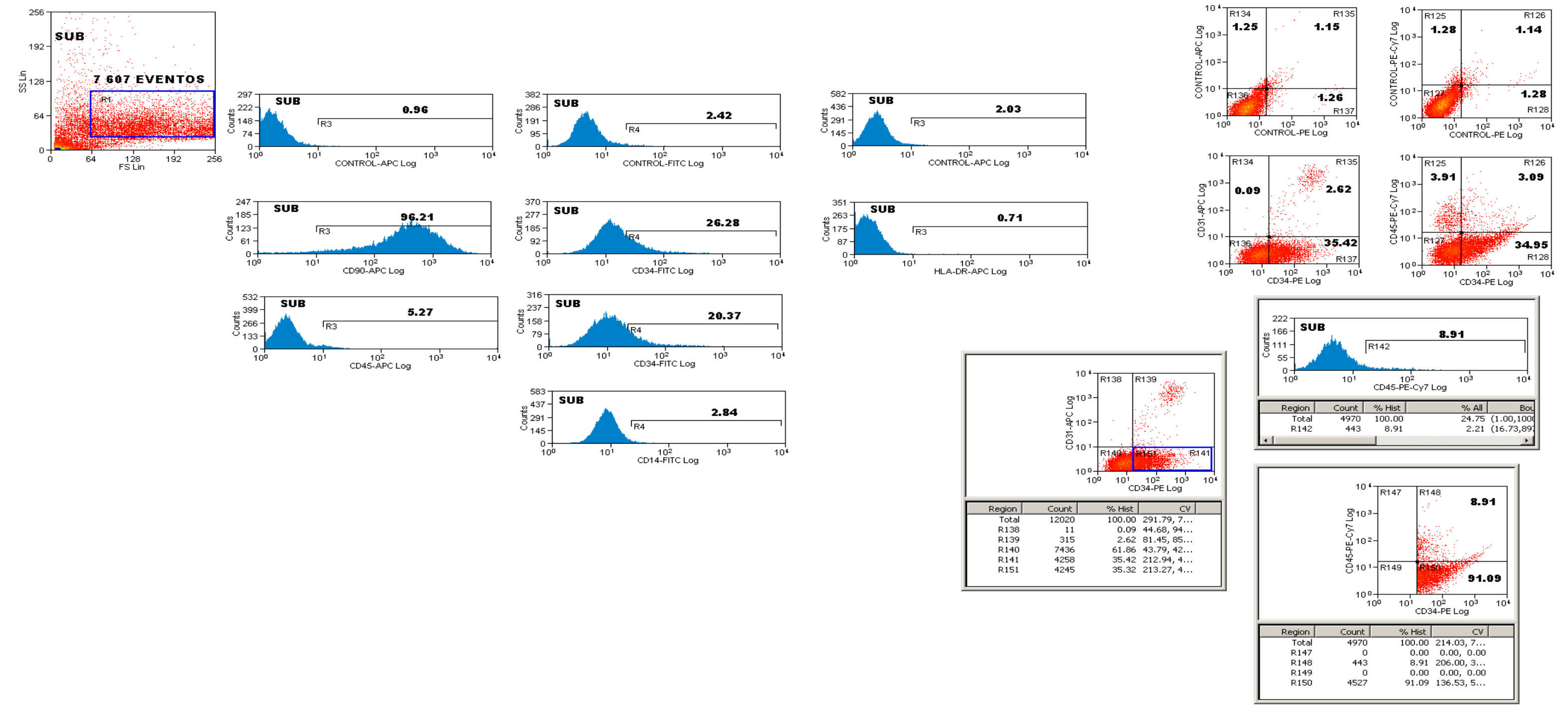

Region	Count	% Hist	CV
Total	12020	100.00	291.79, 7...
R138	11	0.09	44.68, 94...
R139	315	2.62	81.45, 85...
R140	7436	61.86	43.79, 42...
R141	4258	35.42	212.94, 4...
R151	4245	35.32	213.27, 4...

Region	Count	% Hist	% All	Bou
Total	4970	100.00	24.75	(1.00,100
R142	443	8.91	2.21	(16.73,89

Region	Count	% Hist	CV
Total	4970	100.00	214.03, 7...
R147	0	0.00	0.00, 0.00
R148	443	8.91	206.00, 3...
R149	0	0.00	0.00, 0.00
R150	4527	91.09	136.53, 5...

SUBJECT6: TAT-ASCs

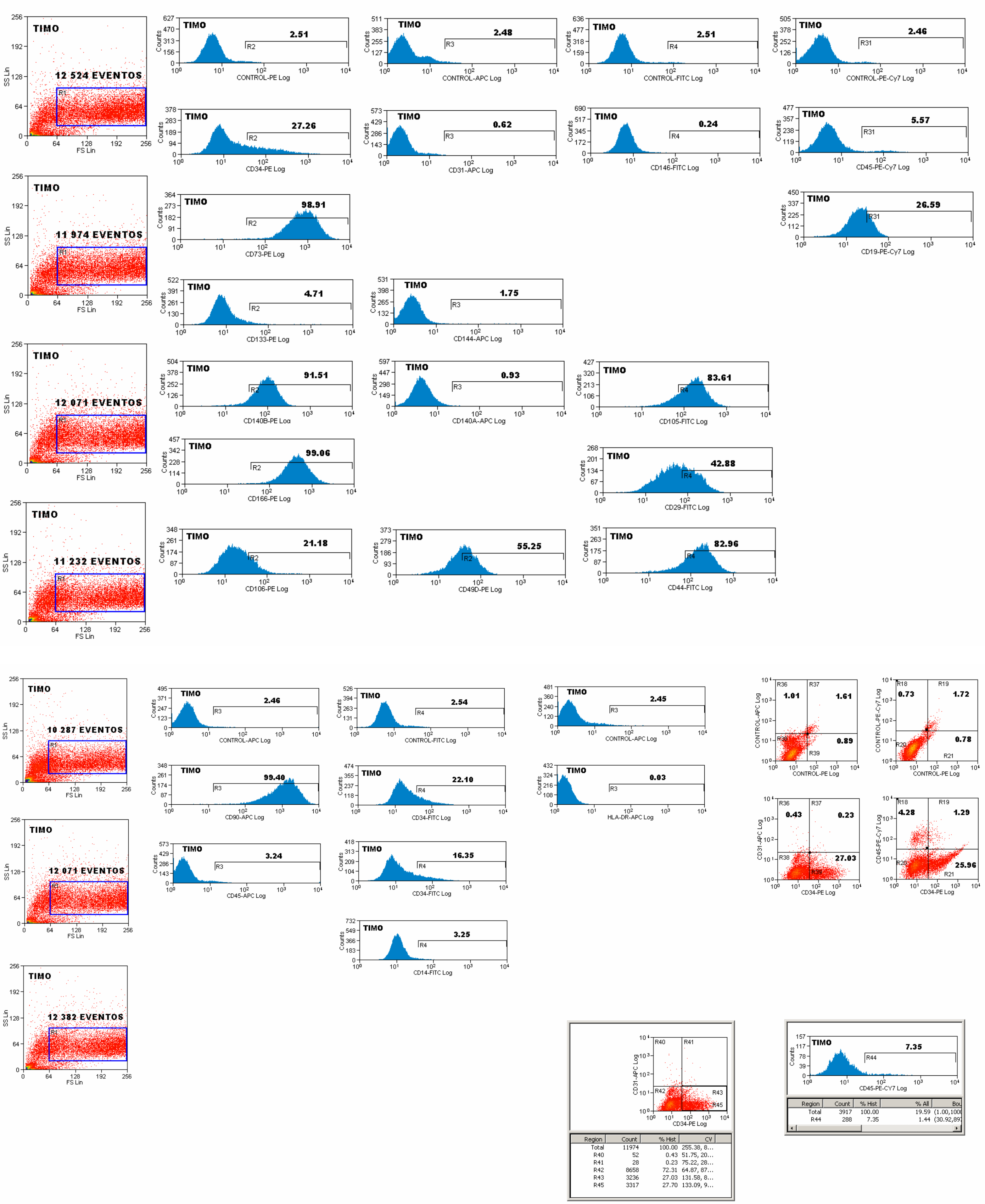

SUBJECT6: SAT-ASCs

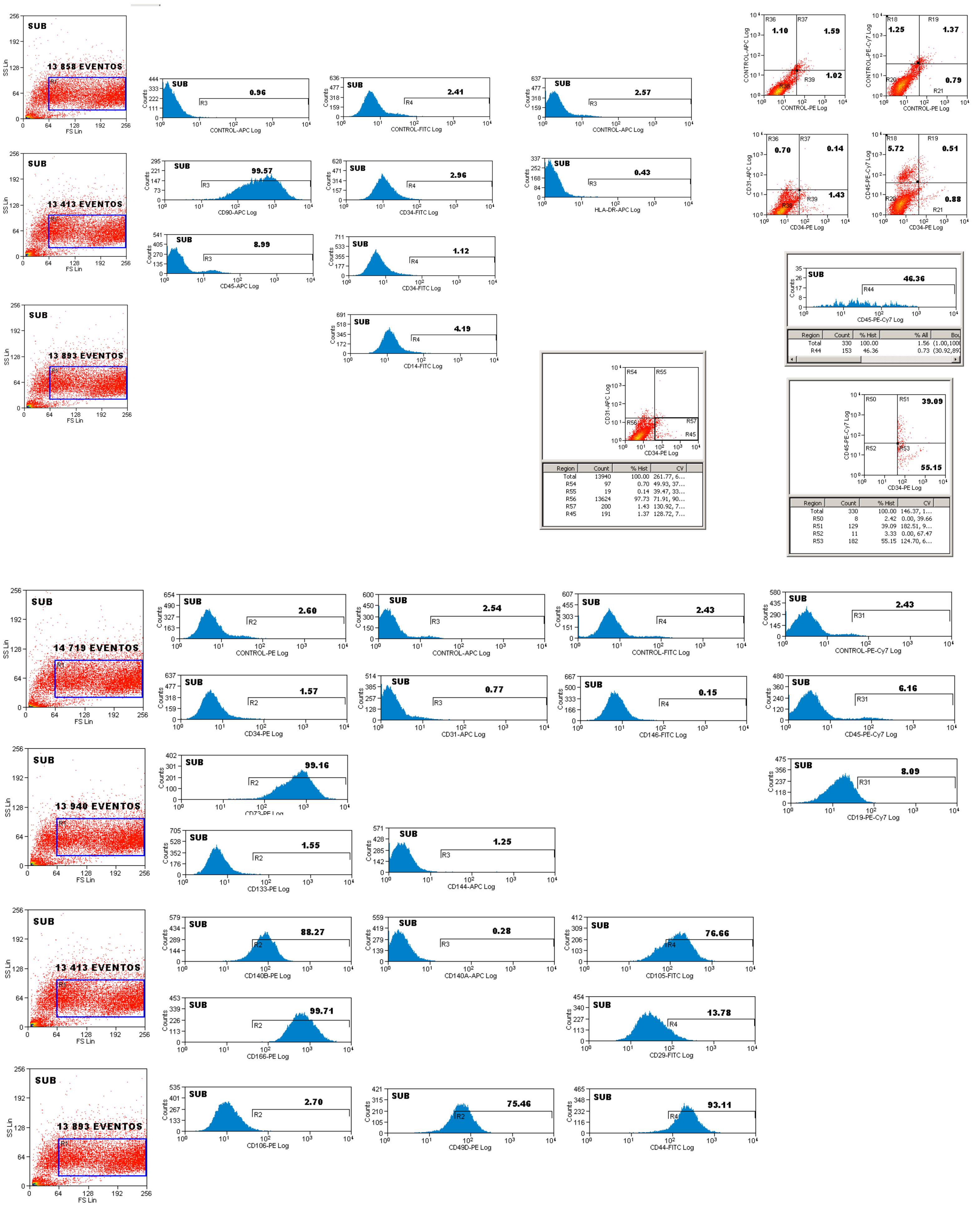

Region	Count	% Hist	% All	Bou
Total	330	100.00	1.56	(1.00,1000
R44	153	46.36	0.73	(30.92,89

Region	Count	% Hist	CV
Total	13940	100.00	261.77, 6...
R54	97	0.70	49.93, 37...
R55	19	0.14	39.47, 33...
R56	13624	97.73	71.91, 90...
R57	200	1.43	130.92, 7...
R45	191	1.37	128.72, 7...

Region	Count	% Hist	CV
Total	330	100.00	146.37, 1...
R50	8	2.42	0.00, 39.66
R51	129	39.09	182.51, 9...
R52	11	3.33	0.00, 67.47
R53	182	55.15	124.70, 6...
