# Supplementary material for: Myocardial Ischemic Subject’s Thymus Fat: A Novel Source of Multipotent Stromal Cells
Source: PLoS One. 2015 Dec 10;10(12):e0144401. doi: 10.1371/journal.pone.0144401 (PMC4675557; doi:10.1371/journal.pone.0144401)
Supplement: S1 Table — FITC: fluorescein isothiocyanate. PE: phycoerythrin. PE-CyTM7: tandem fluorochrome that combines phycoerythrin and cyanine dye. APC: allophycocyanin. (DOC) [file pone.0144401.s002.doc]

**Table S1**

| **MARKERS** | **FLUORO CHROME** | **CLONE** | **ISOTYPE** | **CONCENTRATION** | **LABORATORY** | **REF** |
| --- | --- | --- | --- | --- | --- | --- |
| CD14 | FITC | M5E2 | Mouse IgG2a, k | 20 µl / test | BD Pharmingen | 555397 |
| CD19 | PE-Cy^TM^7 | SJ25C1 | Mouse IgG1, k | 5 µl / test | BD Pharmingen | 557835 |
| CD45 | PE-Cy^TM^7 | HI30 | Mouse IgG1, k | 5 µl / test | BD Pharmingen | 557748 |
| CD73 | PE | AD2 | Mouse IgG1, k | 20 µl / test | BD Pharmingen | 550257 |
| CD34 | FITC | 4H11 | Mouse IgG1, k | 5 µl (0.5 µg) / test | eBioscience | 11-0349-42 |
| CD29 | FITC | TS2/16 | Mouse IgG1 | 20 µl (0.25 µg) / test | eBioscience | 11-0299-41 |
| CD31 | APC | WM-59 | Mouse IgG1, k | 20 µl (0.5 µg) / test | eBioscience | 17-0319-71 |
| CD90 | FITC | 5E10 | Mouse IgG1, k | 20 µl (1.0 µg) / test | eBioscience | 11-0909-71 |
| CD49D | PE | 9F10 | Mouse IgG1, k | 5 µl (0.125 µg) / test | eBioscience | 12-0499-71 |
| CD106 | PE | STA | Mouse IgG1, k | 20 µl (0.125 µg) / test | eBioscience | 12-1069-71 |
| CD133 | PE | TMP4 | Mouse IgG1, k | 5 µl (0.25 µg) / test | eBioscience | 12-1338-41 |
| CD144 | APC | 16B1 | Mouse IgG1 | 20 µl (0.25 µg) / test | eBioscience | 17-1449-73 |
| CD146 | FITC | P1H12 | Mouse IgG1 | 20 µl (0.25 µg) / test | eBioscience | 11-1469-41 |
| CD44 | FITC | DB105 | Mouse IgG1 | Recommended antibody dilution:  1:11 for up to 10^7^ cells/100 µL | Miltenyi | 130-095-195 |
| HLA-DR | APC | AC122 | Mouse IgG2a |  | Miltenyi | 130-095-297 |
| CD105 | APC | 43A4E1 | Mouse IgG1 |  | Miltenyi | 130-094-926 |
| CD140A | APC | PRa292 | Mouse IgG1 | Recommended concentration:  10 µL/10^6^  cells | RD Systems | FAB1264A |
| CD140B | PE | PR7212 | Mouse IgG1 |  | RD Systems | FAB1263P |
| CD166 | PE | 105902 | Mouse IgG | 35 µg / mL | RD Systems | FAB6561P |
